# A flexible statistical model for alignment of label-free proteomics data – incorporating ion mobility and product ion information

**DOI:** 10.1186/1471-2105-14-364

**Published:** 2013-12-16

**Authors:** Ashlee M Benjamin, J Will Thompson, Erik J Soderblom, Scott J Geromanos, Ricardo Henao, Virginia B Kraus, M Arthur Moseley, Joseph E Lucas

**Affiliations:** 1 Institute for Genome Sciences and Policy, Duke University Medical Center, Durham, North Carolina, USA; 2 Waters Corporation, Milford, Massachusetts, USA; 3 Department of Medicine, Duke University Medical Center, Durham, North Carolina, USA; 4 Department of Statistics, Duke University, Durham, North Carolina, USA; 5 Quintiles, Durham, North Carolina, USA

**Keywords:** Proteomics, Ion mobility, Data alignment, Matching, Product ions

## Abstract

**Background:**

The goal of many proteomics experiments is to determine the abundance of proteins in biological samples, and the variation thereof in various physiological conditions. High-throughput quantitative proteomics, specifically label-free LC-MS/MS, allows rapid measurement of thousands of proteins, enabling large-scale studies of various biological systems. Prior to analyzing these information-rich datasets, raw data must undergo several computational processing steps. We present a method to address one of the essential steps in proteomics data processing - the matching of peptide measurements across samples.

**Results:**

We describe a novel method for label-free proteomics data alignment with the ability to incorporate previously unused aspects of the data, particularly ion mobility drift times and product ion information. We compare the results of our alignment method to PEPPeR and OpenMS, and compare alignment accuracy achieved by different versions of our method utilizing various data characteristics. Our method results in increased match recall rates and similar or improved mismatch rates compared to PEPPeR and OpenMS feature-based alignment. We also show that the inclusion of drift time and product ion information results in higher recall rates and more confident matches, without increases in error rates.

**Conclusions:**

Based on the results presented here, we argue that the incorporation of ion mobility drift time and product ion information are worthy pursuits. Alignment methods should be flexible enough to utilize all available data, particularly with recent advancements in experimental separation methods.

## Background

### Label-free proteomics

In a standard “bottom-up” proteomics experiment, proteins are first digested into peptides by a proteolytic enzyme. Peptides in this mixture are then physically separated by Chromatography, often Liquid Chromatography (LC). Eluting peptides are converted to gas phase ions, which are separated in a Mass Spectrometer (MS) by mass-to-charge ratio, and the relative abundance of each ion is measured by a detector. LC-MS experiments utilize a single mass analyzer, resulting in a retention time, mass-to-charge ratio, and intensity for each analyte. In LC, tandem MS experiments, or LC-MS/MS, select precursor ions are further fragmented into product ions, resulting in an additional level of information for each peptide ion. The product ions are analyzed to determine a peptide sequence, which is used to identify the parent protein. A recent variation of LC-MS/MS - Data Independent Acquisition (DIA) - generates product ions for virtually every precursor ion, providing tremendous utility for quantification and identification in a single data set. Examples of DIA include SWATH [1] and MS^E^[2]. In MS^E^, precursor ions enter a collision cell, rapidly alternating between high and low kinetic energy states. This “high-low switching” fragmentation enables the measurement of both precursor and product ions in a single experiment. An even more recent DIA approach to bottom-up proteomics experiments - HDMS^E^ – incorporates Ion Mobility (IM) spectrometry, an additional separation of peptide ions after LC, and before MS^E^. IM spectrometry separates ionized peptides based on charge and three-dimensional cross-sectional area.

### Label-free proteomics data processing

**Figure 1:**
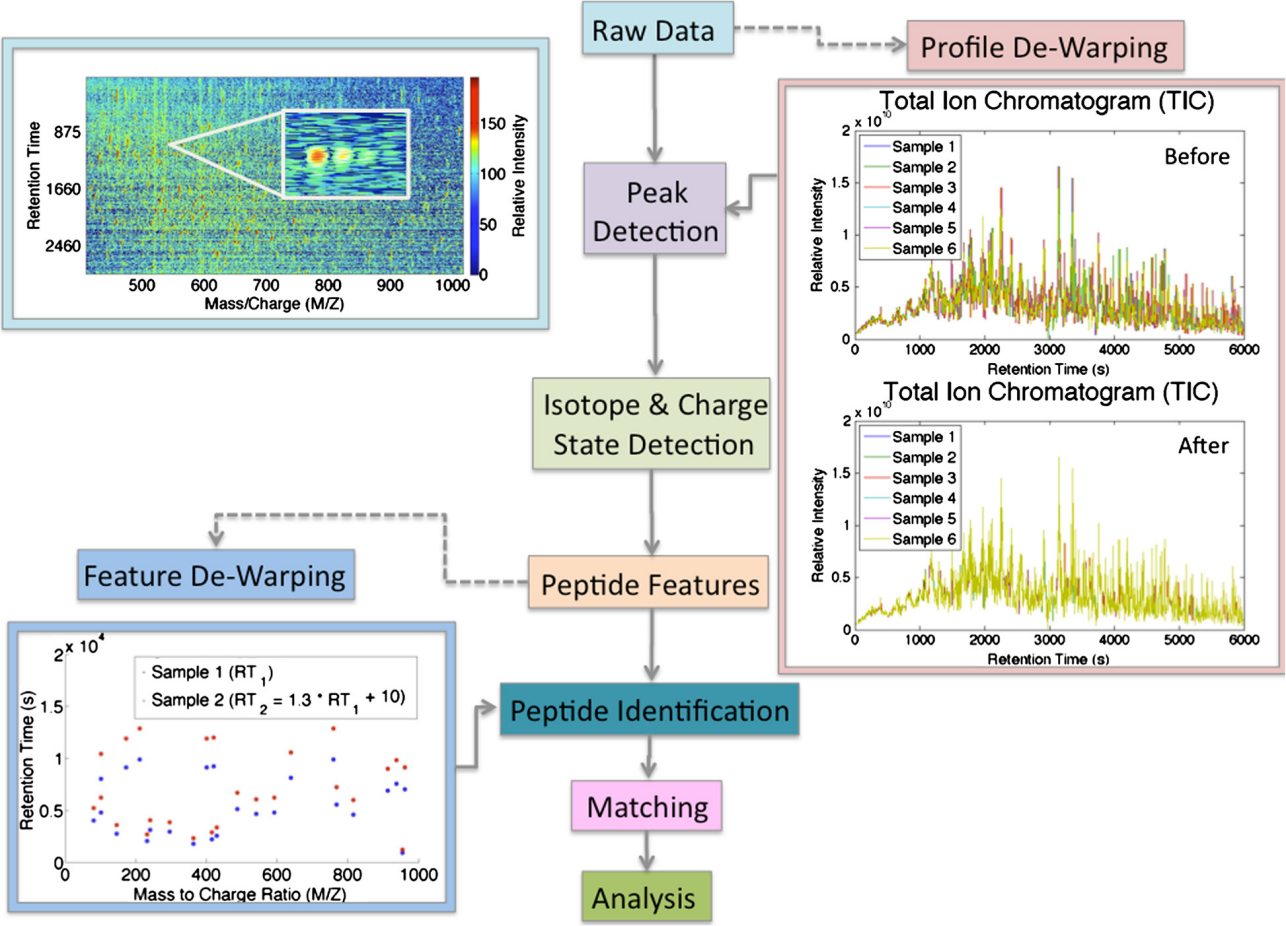
**Processing label-free proteomics data.** Raw data contains peptide and noise peaks, with each peptide presenting as several peaks due to multiply charged ions and the presence of different isotopes (i.e. the presence of one or more _13_C). Ideally, all true peptide peaks are found and combined into a single peak per peptide (though different charge states are often left as multiple features). A common peak detection and de-isotoping technique is to repeatedly determine the most intense peak in the dataset, and determine the charge state and isotopic distribution from the frequency and intensity of the neighboring peaks. The LC retention times and elution order of peptides often shift between runs. The process of correcting these distortions to allow accurate matching across runs is called de-warping. De-warping is either performed on raw profile data, or feature data. After de-warping, peptide features are matched across samples.

Several data processing steps are required to elucidate individual peptide intensities from raw label-free proteomics data. A typical data processing pipeline for a label-free proteomics experiment with multiple samples is illustrated in Figure 1. Peptide peaks must be discerned from noise, charge states determined, and isotopic distributions identified and often combined into peptide features. Further details regarding current peak detection, de-isotoping, and charge state detection methods are described in Dowsey et al. [3] and Zhang et al. [4]. The LC retention times and elution order of peptides often shift between runs. Such variations in retention time are typically called warp. The process of correcting these distortions to allow accurate matching across runs is called de-warping. Many de-warping methods exist, performing linear or non-linear (or both) corrections of two or more samples [5]. This de-warping step is either performed on raw profile data (prior to or independent of peak detection and de-isotoping), or on feature data (detected peptide features). After generation of a peptide feature set, peptide identifications are made wherever possible, and intensity measurements of both identified and unidentified peptides are grouped across runs, creating a peptide-by-sample intensity array for subsequent analyses. It should be noted that the order of data processing steps may vary within different pipelines. These data processing steps pose significant computational challenges, and are thought to be the source of much irreproducibility. This was illustrated by a recent test study by Bell et al. [6]. In the study, a sample of 20 proteins was distributed to 27 different labs, experimentally analyzed, and subjected to a variety of computationsl data-processing methods. There were significant discrepancies in reported proteins, however, all raw data was sufficient to identify all 20 proteins when centrally re-processed.

### Label-free proteomics data alignment

We focus on the problems of simple data de-warping, and matching peptide intensities across multiple high-throughput proteomics runs - a combined processing step we call alignment. Our analysis emphasizes the data matching step. Accurate alignment is essential in large-scale proteomics experiments, particularly in biomarker discovery where the comparative nature of these studies require intensities of the same peptide to be compared across samples [7,8]. In addition, accurate matching across samples can increase identifications as information can be leveraged from all individual runs [9]. The complexity of biological samples, however, poses significant computational challenges for both data alignment and peptide identification. Most samples contain tens of thousands of peptides and measurements often reflect “overlapping” peptides, or co-eluting peptides having nearly the same mass-to-charge ratio. These overlapping peptides complicate and often prevent identification. However, recent experimental advancements provide additional separation information that has not yet been leveraged in data alignment - namely comprehensive product ion information with DIA, and IM drift times with HDMS^E^. Product ions have been used extensively in database matching for peptide identification [10,11], but are not widely used in proteomics data alignment. Matching across samples is typically performed using experimentally measured and inferred characteristics of each peptide feature. Measured characteristics include precursor ion retention time, mass-to-charge ratio, and intensity. Depending on the experimental methods, some or all peptide features may have additional measured characteristics including the intensities, mass-to-charge ratios, and retention times of product ions. In DIA experiments, virtually every peptide feature has measured product ion data. In HDMS^E^ experiments, all precursor and product ions have a measured IM drift time as well. Inferred characteristics include charge state for nearly every peptide feature, and an amino acid sequence for some peptide features. Modern high-throughput proteomics experiments offer a great deal of information, not all of which is currently utilized in data processing - specifically in data alignment steps.

### Previous alignment approaches

Matching methods utilize various aspects of the data to group peptide measurements across samples. Previously utilized characteristics include retention time, mass-to-charge ratio, intensity, and amino acid sequence. Incorporating additional data provides a higher degree of specificity when making matches [5]. The majority of existing alignment techniques utilize mass-to-charge ratio and retention time information. Such methods include SpecArray [12], AMT tag approaches [13], Xalign [14], MZmine [15], msInspect [16], XCMS [17], PETAL [18], OpenMS [19,20], apLCMS [21], and MZmine2 [22]. Semi-supervised approaches such as PEPPeR [23] and a method by Fischer et al. [24], take advantage of existing MS/MS peptide identifications. More recent alignment methods by Tang et al. [25] and Zhang et al. [26] utilize peptide intensity along with mass-to-charge ratio and retention time. SuperHirn [27] indirectly incorporates intensity information during multiple alignments by performing pairwise alignments in a specific order based on LC-MS similarity and intensity correlations.

In addition to utilizing novel data characteristics, our method is designed to avoid common pitfalls of other approaches including static distance cutoffs, elution order assumptions, and the selection of a single reference sample. Methods should not rely on the fact that peptides always elute in the same order from the LC column as this is a false assumption and can introduce matching errors [28-30]. Multiple alignment methods often require a reference run to which all other runs are aligned. While this approach is successful for the de-warping step, choosing a single reference run for the matching step can be problematic. If the measurement variability is high in the reference sample, this can result in incorrect matches. We present a novel statistical alignment method that corrects for linear global variation, is not restricted by static distance cutoffs, and has the ability to utilize retention time, mass-to-charge ratio, peptide identifications, and previously ignored aspects of proteomics data - ion mobility drift times, and product ion data. Our method is an adapted Bayesian Dirichlet Process Gaussian Mixture Model (DPGMM) [31,32], adding sample-specific shift and scale parameters. The proposed method is also easily extensible to incorporate additional dimensions, such as a second LC separation. We present the results of our alignment model on various datasets, comparing alignment accuracies with the inclusion of various data characteristics.

## Results

### *E. coli* lysate data

To assess both the performance of our alignment method, and the utility of various data characteristics, we aligned technical replicates of *E. coli* lysate data. Three technical replicates of 500ng of *E. coli* lysate were analyzed with Waters MS^E^ and HDMS^E^. The MS^E^ data was used to compare our alignment method with PEPPeR [23], and the feature matching functionality of OpenMS [19,20]. The PEPPeR PeakMatch module was downloaded from GenePattern [33] and run locally using default parameter settings. Similarly, the MapAlignerPoseClustering, and FeatureLinkerUnlabeled functions of OpenMS version 1.11.0 were run using default parameter settings to align peptide features of MS^E^ data. The HDMS^E^ data was used to compare the results of our alignment method when utilizing various data characteristics. Table 1 shows the four different combinations of data characteristics utilized for alignment. We assess alignment performance using held-out peptide identifications resulting from product ion spectra.

**Table 1:** Alignments and data utilization

**Alignment**	**Parent ion**	**Parent ion**	**Parent ion**	**Production**
**name**	**Monoisotopic**	**peak centroid**	**drift time**	**profile**
	**mass-to-charge**	**time**		
	**ratio**			
MZ-RT	✓	✓		
MZ-IM	✓		✓	
MZ-RT-IM	✓	✓	✓	
MZ-RT-IM-HE	✓	✓	✓	✓

The alignment type names and data utilized that were compared in the analysis.

**Figure 2:**
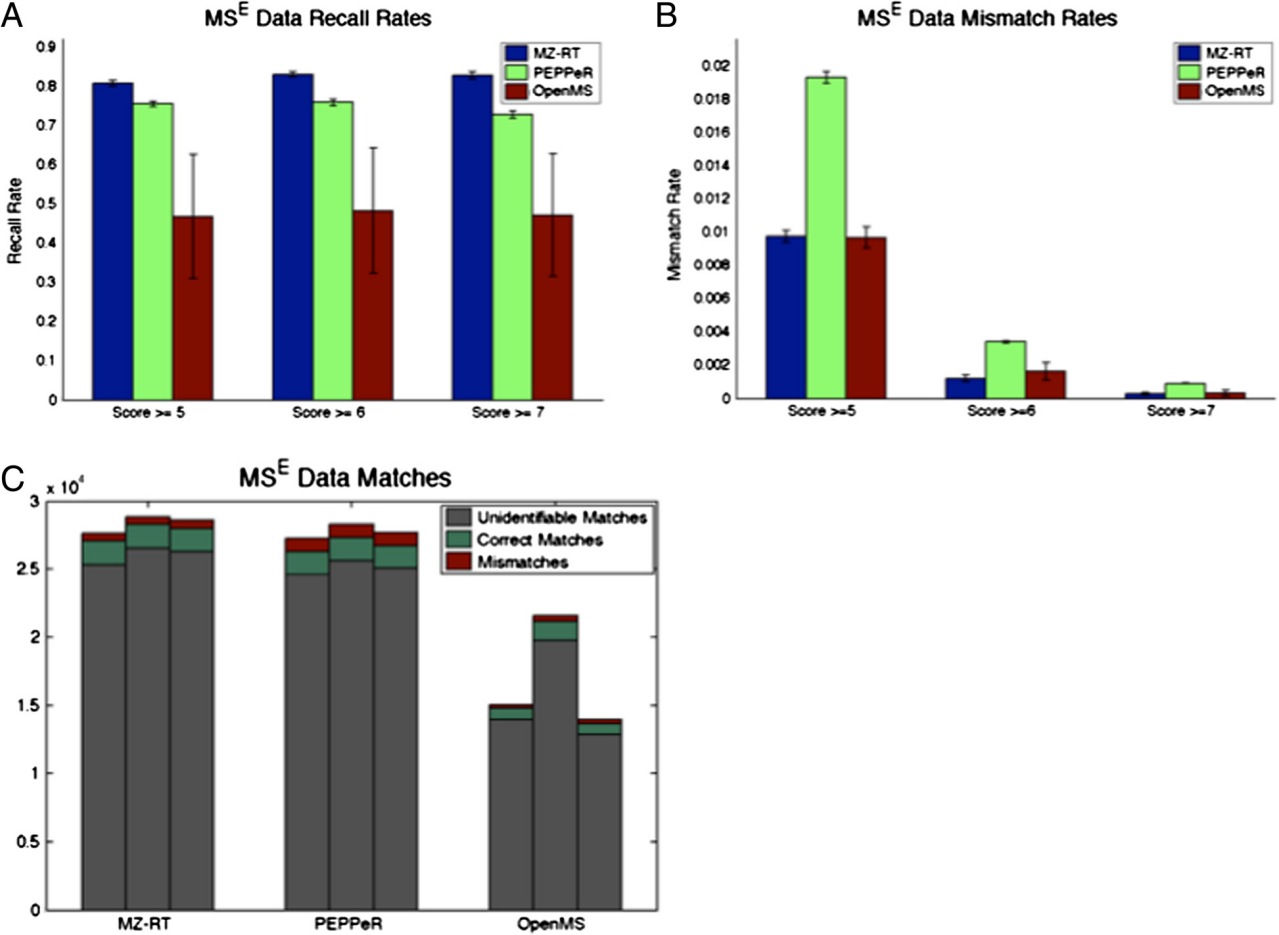
**Comparison with PEPPeR and OpenMS.** Panel **A** shows the recall rates for the MS^E^*E. coli* lysate data, considering identifications having peptide score 5, 6, and 7 or greater, for our method, OpenMS, and PEPPeR. Panel **B** shows the mismatch rate for the MS^E^*E. coli* lysate data, considering identifications having peptide score 5, 6, and 7 or greater. The mismatch rate is computed as the number of mismatches (pairwise match with conflicting identifications) divided by the total matches. Panel **C** shows a bar plot of all correct, incorrect and unidentifiable matches for each method. Unidentifiable matches are pairwise matches where neither peptide has a putative peptide sequence, and so the accuracy cannot be inferred.

The MS^E^*E. coli* lysate data was aligned using PEPPeR, OpenMS, and our alignment method, utilizing precursor ion mass-to-charge ratio and retention time (MZ-RT). Each alignment method was provided with the same 15 “given matches” to initialize model hyperparameters – the 15 identifications shared by all three replicates having the highest average ProteinLynx Global SERVER (PLGS) Identity^E^[34] peptide score. When assessing alignment performance, we examine matches between each replicate pair – ID0821901 vs. ID0821902, ID0821901 vs. ID0821903, and ID0821902 vs. ID0821903. Correct matches occur when two identified peptides shared between the pair of technical replicates are aligned. Mismatches occur when two identified peptides with conflicting identifications are aligned. Since PEPPeR allows multiple peptides from a single sample to be present in a “cluster”, there may be more than one identification from a single replicate. In these cases, we counted a correct match if shared identifications were present in the same cluster. Similarly, we counted a mismatch if conflicting identifications were present in the same cluster. As there are varying levels of confidence in peptide identifications, we present the results of each alignment method considering identifications having a PLGS Identity^E^[34] peptide score of five, six, and seven or greater. Panels A and B of Figure 2 show the recall rate and the mismatch rates, respectively, for each alignment method. As PEPPeR does not directly report match confidence, recall and mismatch rates were computed from all reported matches for each method. We see that our alignment method obtains significantly higher recall rates than OpenMS FeatureLinker and PEPPeR, for identified peptides of each confidence level. When comparing the mismatch rates, computed as the number of mismatches divided by the total matches, we see that our method obtains mismatch rates comparable with OpenMS, while PEPPeR obtains significantly higher mismatch rates, particularly at lower peptide scores. It should be noted that this mismatch rate is not a false positive rate. The total match count includes many matches which cannot be identified as correct or incorrect, as neither peptide has a putative peptide sequence. When examining the total match counts in Panel C of Figure 2, we see that our method obtains match counts comparable to PEPPeR, and significantly more matches than OpenMS.

**Figure 3:**
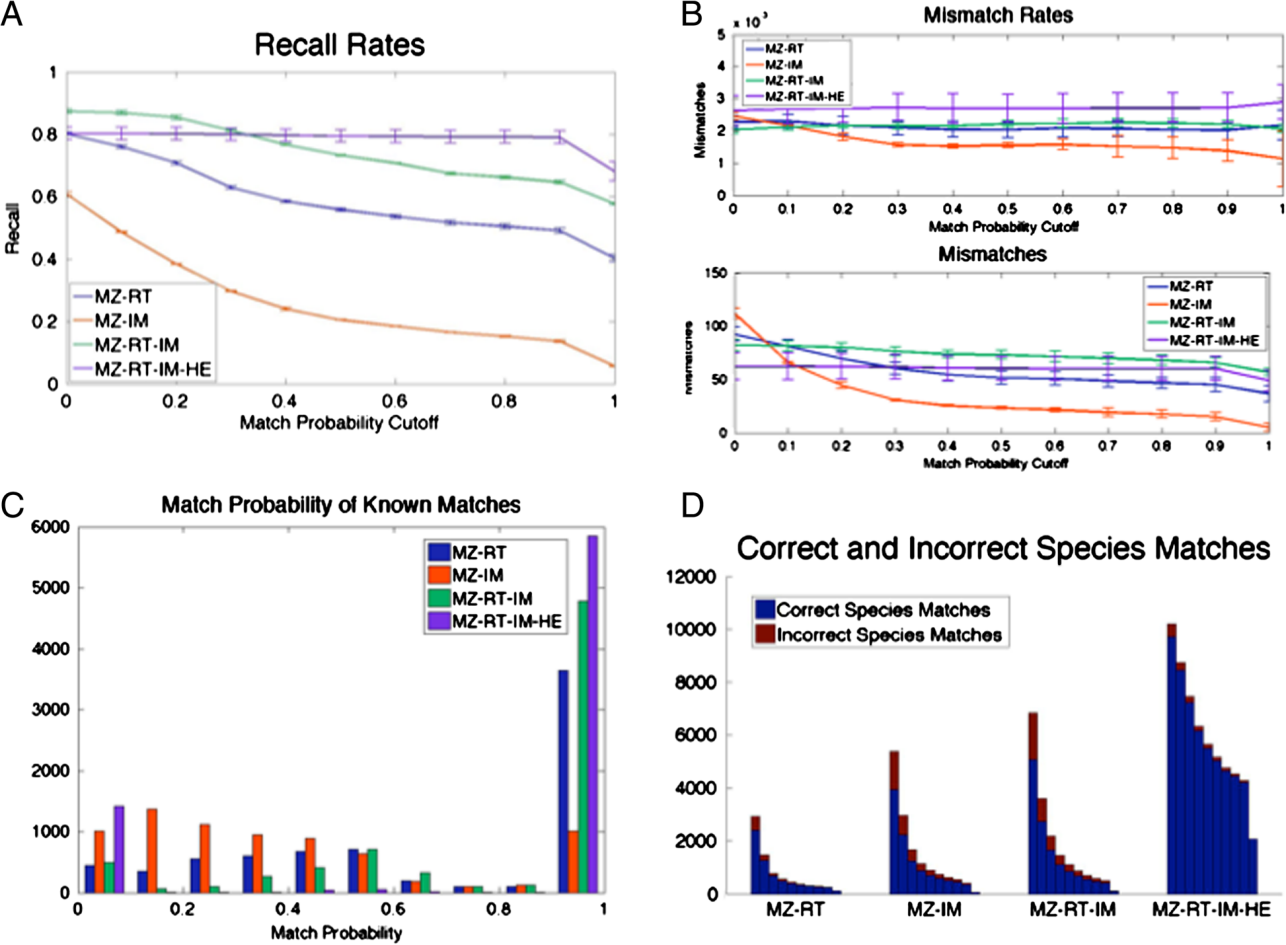
**Alignment results using various data characteristics.** Panel **A** shows the recall rates for the HDMS^E^*E. coli* lysate data, considering identifications having peptide score 5 or greater, for each of the four alignments across a range of match probability cutoffs. Panel **B** shows the number and rate of mismatches for the HDMS^E^*E. coli* lysate data, considering identifications having peptide score 5 or greater, obtained for each of the four alignments, across a range of match probability thresholds. Panel **C** shows a histogram of the match probabilities of all shared identifications having peptide score 5 or greater, for each of the four alignments of the *E. coli* lysate data. For the decoy analysis, Panel **D** shows the number of matches made to the correct species, and the number of matches made to the incorrect species for each of the four alignments, across a range of match confidence. Each group of stacked bars indicates the number of correct and incorrect matches for the indicated alignment type, for increasing match confidence thresholds from 0.1 to 1 in 0.1 intervals.

The HDMS^E^*E. coli* lysate data was aligned using different data characteristics to compare their utility for alignment. As with the MS^E^ data, each alignment was provided with the same 15 “given matches” to initialize model hyperparameters and the remaining identifications were used to assess alignment performance. We present the results of each alignment only considering identifications having a PLGS Identity^E^[34] peptide score of five or greater. Results at additional peptide score thresholds are provided in Additional file 1. Comparing the HDMS^E^ alignments with various data combinations informs about the utility of each dimension in data alignment. We examine matches between each replicate pair – ID0822001 vs. ID0822002, ID0822001 vs. ID0822003, and ID0822002 vs. ID0822003. A match across two samples occurs when a measured peptide feature from each sample is assigned to the same latent peptide feature. Panels A and B of Figure 3 show the recall rate and the rate and number of mismatches, respectively, for each of the four alignments. We performed a series of two-sample t-tests assuming equal variance for the recall rates and mismatches to assess differences between alignments. When examining the results of the two-dimensional parent ion alignments, MZ-RT and MZ-IM, we see that the MZ-RT alignment obtains significantly higher recall rates for matches of all confidence levels. Utilizing all parent ion data characteristics obtained via HDMS^E^ with a three-dimensional alignment (MZ-RT-IM) results in significantly higher recall rates than the two-dimensional alignments, with a small increase in mismatches from the MZ-RT alignment, and a significant increase in mismatches from the MZ-IM alignment. When including product ion profiles, we see significant increases in recall rates, particularly with more confident matches. We see an insignificant increase in mismatches from the MZ-RT and MZ-RT-IM alignments. The alignment including product ion profiles results in much more confident matches overall. It should be noted that the results presented for the *E. coli* lysate analysis assume that all peptide identifications having an Identity^E^[34] peptide score 5 or greater are correct. We also assume that a match of two peptides differing only by a leucine vs. isoleucine amino acid call or by amino acid order in the peptide sequences, still represents an mismatch. The resulting p-values across the range of match probability stringencies are available in Additional file 1. We also examined the match probabilities of all shared identifications – the peptides that should be matched - between the replicates, for each of the four alignments. Histograms of the match probabilities are shown in Panel C of Figure 3. We see many more confident match probabilities (near 1) of the shared identifications for the MZ-RT-IM when comparing to both two-dimensional alignments. When including product ion profiles with the MZ-RT-IM-HE alignment, we also see an increase in confident match probabilities, with a migration of all intermediate match probabilities to near-zero.

### Human with *E. coli* lysate decoy

In order to assess alignment performance without experimental identification bias, we aligned technical replicates of *E. coli* lysate data with a Human plasma decoy, and technical replicates of Human plasma with an *E. coli* lysate decoy. One technical replicate of the *E. coli* lysate sample was aligned with another *E. coli* lysate technical replicate combined in silico with a decoy Human plasma sample. To combine the samples, we append the human plasma peak list onto the peak list of one of the *E. coli* replicates. The Human plasma was a pooled sample from 20 individuals, run with 2 technical replicates. Similarly, one replicate of the human plasma was aligned with another plasma technical replicate combined with an *E. coli* lysate sample. As with the *E. coli* lysate data, we performed the four different alignments described in Table 1. Each alignment was provided with the same 15 “given matches” – the 15 identifications shared by the technical replicates, and having the highest average PLGS Identity^E^[34] peptide score. To assess alignment performance, we determine the proportion of incorrect species alignments. This provides a false discovery rate that avoids experimental identification bias - assuming the set of shared peptides across species is negligible [35]. Figure 3, Panel D shows the correct and incorrect species match counts for each of the four alignments. We see that the MZ-RT-IM and MZ-IM alignments yield similar results, while the MZ-RT alignment obtains fewer incorrect species matches, but fewer matches overall. The MZ-RT-IM-HE alignment obtains the least incorrect species matches, and many more correct species matches at reasonably confident match levels. The results of the inverse decoy analysis (aligning technical replicates of *E. coli* lysate with a Human plasma decoy) are available in Additional file 1: Figure S7.

### Hepatitis-C and osteoarthritis data

To illustrate the utility of aligning datasets obtained from two different tissues, we aligned MS^E^ serum samples of Hepatitis-C patients with MS^E^ human urine samples from an Osteoarthritis cohort using peptide ion mass-to-charge ratio and retention time. Peptide identifications from the urine samples were carried over to the serum via alignment. We examined the 500 peptides having the most significant differential expression with a phenotype of interest – Hepatitis-C treatment response. Significance was assessed with two-sample t-tests assuming equal variance, on peptide intensities that were log-transformed, mean-centered by sample, and standardized by peptide. We explored the inferred identifications that were carried over via alignment from urine, in order to identify potential biomarkers. Specifically, we looked at the previously unidentified peptides exhibiting significant differential expression having an inferred identification from the alignment results. We analyzed these inferred identifications using GATHER [36] and DAVID [37] to search for functional and pathway enrichment. The lists of proteins from these inferred identifications and their GATHER and DAVID analysis results are available in the Additional file 1. The inferred identifications of peptides exhibiting differential expression for Hepatitis-C treatment response totaled 42 corresponding proteins. These proteins were significantly enriched for defense response (GO0006952 - 15 proteins), immune response (GO0006955 – 15 proteins), and response to biotic stimulus (GO0009607 – 14 proteins), having GATHER p-values 1.56e-9, 3.76e-9, and 9.81e-9, respectively. These functional annotations are very much in accordance with what one would expect with a response to viral infection, suggesting that useful identifications were obtained with the alignment. In addition, the genes encoding 6 of these proteins were located at 14q32, indicating significant chromosome location enrichment with GATHER p-value 1.27e-5. This is the location of the immunoglobulin heavy locus – a region containing genes encoding the heavy chains of antibodies. Differential expression of genes in this region is also in accordance with what one would expect for HCV treatment response.

## Discussion

We have developed a novel method for label-free proteomics data alignment that incorporates aspects of the data ignored by other open-source data alignment methods. Our alignment method incorporates ion mobility separation data and MS/MS product ion data. Our results suggest that the inclusion of more data characteristics increases alignment sensitivity, and increases matching robustness.

When comparing to OpenMS, our method obtains significantly higher recall rates, as well as more overall matches. This is likely due to the density of the data we use for comparison, and the matching technique of OpenMS. After the de-warping step, OpenMS makes pairwise matches between samples, or “maps”, if the putative match is the nearest neighbor and the distance to the second-nearest neighbor is significantly greater. This results in low false positive rates, as seen in Panel B of Figure 2. However, in dense datasets this appears to result in lower recall rates as many true matches are not considered. In Panel C of Figure 2, we also observe that for OpenMS, one pairwise combination of technical replicates shows significantly more matches than the other pairwise combinations. This may be the result of selecting a single reference sample to which all other samples are aligned. OpenMS first selects the sample with the most features as the reference. Each remaining sample is then aligned to the reference, estimating a “consensus” with each pairwise alignment. The density and number of features within the consensus increases with each pairwise alignment, resulting in fewer matches meeting the nearest-neighbor criteria at each step. It should be noted that we only evaluated the peptide feature-based functionality of OpenMS as a comparison to our feature-based alignment method. The ability to work with raw data, alignment specificity and ease-of-use of OpenMS are advantageous for many applications.

Our alignment model before the addition of the product ion component is very similar to PEPPeR [23] – both methods are built on the principles of Gaussian Mixture Models. Our results in the MS^E^ comparison (Figure 2) reflect the similarity of our approaches. The three main differences between PEPPeR and our MZ-RT method are the technique for inferring the number of mixture components, PEPPeR’s splitting of the data by charge state, and our constraint allowing only one measured peptide per sample in a given mixture component. We chose to ignore charge state information to avoid propagation of errors from earlier data-processing steps, although alignments can easily be stratified by charge state with our method.

Figure 3 illustrates the significant improvements resulting from the inclusion of ion mobility and product ion data, while maintaining low levels of mismatches. In addition, the inclusion of more data – particularly the product ion profile information – results in increased confidence and robustness of alignment matching. We note that none of the alignments reach a recall rate of 1. This is likely due to to the tendency of our method to generate new latent peptide when a confident match to an existing latent peptide does not exist. This same behaviour avoids large numbers of false positive matches.

In our decoy experiment, we observed that the addition of product ion data results in a dramatic decrease in false matches, this is likely due to the lack of confounding product ion assignments to precursor ions as the decoy data is a separate experiment. However, if one were utilizing product ion data to align measurements to an AMT tag-like database, we would expect a comparable situation. These results also speak to the importance of accurate product ion to precursor ion assignment in DIA – if peptides were well separated experimentally and accurate product ion assignments were made, alignment accuracy would increase dramatically.

Aligning data from different experiments can actually yield additional identifications, as illustrated by the alignment of human urine data to human serum. Due to the diverse protein composition of different types of samples, specific peptides may be identified more easily in certain types of samples. It is worth noting that this behavior is much like spectral library searching [38], because surrounding peptides will not confound the product ion assignments to precursor ions. We show that the alignment of data from different tissues (even when only utilizing precursor ion data) has utility for inferring peptide identifications. If this were extended to a database and data from many tissues were used to update the database, it could have a comprehensive identification set of measured peptides, and be utilized as an additional resource or replacement for de novo identification. This is particularly useful in biomarker analyses when performing a label free experiment for an initial analysis, and then identifying proteins of interest for a subsequent targeted analysis. Also, the addition of product ion data will provide more confident alignments, and thus more confidence in identifications that may be carried over.

Although we argue that the incorporation of product ion data can result in more matches of increased confidence, it should be noted that the method in which these data are incorporated has importance. If the presence of additional product ions, or the lack of product ions is highly penalized, alignments are likely to obtain fewer matches due to the variability in measurement of product ions. Conversely, if differences in product ions are not penalized enough, alignments are likely to obtain more matches, and more mismatches – particularly because nearby peptides with respect to mass-to-charge ratio, retention time and drift time, will be those with incorrect, but similar product ion profiles. When incorporating product ion data, researchers should consider the penalization of extra and missing product ions within the data being aligned. We found that similarity functions based on sums rather than products worked well, specifically, the sum of squared differences. Our exploration of other product ion profile similarity functions is described in Additional file 1.

## Conclusions

We have developed a flexible alignment method that effectively incorporates two data characteristics novel to open-source peptide feature matching. Our method provides accurate and robust matches between samples or datasets with measures of confidence. We also show that peptide feature alignment of disparate data sets has utility in biomarker analyses, as the ability to identify certain proteins may vary by tissue. The results presented here provide motivation for further exploration of incorporating additional separation information into proteomics data processing, particularly as experimental advancements are made in the field.

## Methods

### Alignment model

We present a statistical model for the alignment of label-free proteomics data to match peptide features across multiple samples after peak-detection and de-isotoping. Unlike any existing proteomics alignment method, our model has the ability to utilize ion mobility drift time from HDMS^E^ experiments, and product ion spectra from traditional LC-MS/MS Data Dependent Aquisition (DDA), or DIA (MS^E^ or HDMS^E^) experiments, along with the typical parent ion mass-to-charge ratio and retention time - increasing the individuality of each peptide feature and providing a better alignment. At the time of publication, no open-source proteomics file format was capable of storing ion mobility separation data. In order to allow incorporation of this data into our alignment method, we wrote a small data-processing script to read Waters ‘spectrum.xml’ and ‘finalfrag.csv’ files into a Matlab data frame. The Matlab data frame format, a sample configuation file, a sample queue submission file, and the data processing script are available in Additional files 1, 3, 4 and 5, respectively, and can be easily adapted to incorporate any additional separation dimensions similar to ion mobility drift times and liquid chromatography retention times, including retention times from multidimensional LC.

#### Model

We adapt a DPGMM [31,32] by adding sample-specific shift and scale parameters. Gaussian Mixture Models lend themselves well to the problem of proteomics data alignment. Each peptide existing in nature has a theoretical mass-to-charge ratio, retention time, etc. within a specific experimental condition, and is represented in our model as a mixture component. We expect the measurement of a peptide to have the same mass-to-charge ratio, retention time, etc., with two different types of measurement error: systematic error and random error. As with any laboratory experiment, LC-MS/MS data are subject to variability. The LC retention times often shift between runs. Pressure fluctuations, changes in column temperature, column manufacturing differences, and peptide interactions can cause changes in the elution time, and/or the elution order of peptides [13]. The mass-to-charge ratios are also subject to measurement error, albeit to a lesser degree than the LC dimension. We account for systematic error with a global shift and scale. Such a transformation would most likely be the result of variations in LC protocols (total run times), or in the time it takes for the first peptide to elute from the column (gradient delays due to different tubing volumes). The remaining random error is assumed to be a sum of small variations from many independent sources of variation, and therefore have a Gaussian distribution. In addition, Gaussian distributions are closed under linear transformations, allowing straightforward computation of posterior distributions with the addition of the shift and scale parameters. Measurements assigned to the same mixture component, or latent peptide, by the model are considered to be matched.

Seed peptide matches are determined with identified peptide sequences and charge states. To avoid introducing error with incorrect identifications, outliers with respect to mass-to-charge ratio and retention time are discarded. These matches are used to initialize hyperparameters, and remain matched at all iterations of the MCMC. Mixture component assignments are given a Chinese Restaurant Process prior, allowing the addition of a new latent peptide if no suitable match exists. Our model addresses simple linear de-warping of the data, however, any preferred de-warping method may be applied prior to utilizing our algorithm. We first describe the model for peptide-level alignment, and then describe the extension to include productions.

#### Peptide-level model

A sample-specific linear shift (*η*_*d*_) and scale (*β*_*d*_) is used for de-warping. In the formulas that follow, samples or datasets are indexed by *d*, individual measured peptides within a sample are indexed by *i*, and latent (theoretical) peptides are indexed by *j*. As shown in Equation 1, we assume that a measured peptide feature, *x*_*d*,*i*_, is a shifted and scaled noisy measurement of a “true” peptide feature, $z_{c_{d,i}}$. Let *c*_*d*,*i*_ be an indicator variable for the latent peptide assignment of measurement *x*_*d*,*i*_, taking on values *j*=1…*J* where *J* is unbounded.

(1) $$\begin{array}{ll} & x_{d,i}=\eta_{d}+z_{c_{d,i}}\beta_{d}+\varepsilon_{d,i}\; \end{array}$$

(2) $$\begin{array}{ll} & \varepsilon_{d,i}∼\text{Normal}(0,\Sigma )\; \end{array}$$

(3) $$\begin{array}{ll} & z_{c_{d,i}}∼\text{Normal}(\mu_{c_{d,i}},\sigma )\; \end{array}$$

Where *ε*_*d*,*i*_ are the residuals between the measured values (*x*_*d*,*i*_) and the shifted and scaled latent values ($\eta_{d}+z_{c_{d,i}}\beta_{d}$), having a multivariate normal distribution - Equation 2. Let *Σ* be the covariance of these residuals, and let each latent peptide $z_{c_{d,i}}$ be a draw from DPGMM mixture component *c*_*d*,*i*_=1…*J* having mean $\mu_{c_{d,i}}$ and covariance *σ*, as shown in Equation 3. Note that we make the simplifying assumption of shared covariance across all latent peptides. This would suggest that the measured values of each latent peptide show the same variation across the entire mass-to-charge ratio, retention time, and drift time range. We acknowledge that this is not likely the case, although we find this assumption works well in practice. Conjugate priors are used for all model parameters as follows:

(4) $$\begin{array}{ll} & \eta_{d}∼\text{Normal}(a_{d},b_{d})\; \end{array}$$

(5) $$\begin{array}{ll} & \beta_{d}∼\text{Normal}(e_{d},f_{d})\; \end{array}$$

(6) $$\begin{array}{ll} & \Sigma ∼\text{Inverse}-\text{Wishart}(s,t)\; \end{array}$$

(7) $$\begin{array}{ll} & \mu_{j}∼\text{Normal}(\lambda ,r)\; \end{array}$$

(8) $$\begin{array}{ll} & \sigma ∼\text{Inverse}-\text{Wishart}(g,h)\; \end{array}$$

Normal priors are assigned to the shift and scale parameters as shown in Equations 4 and 5. The seed matches, for each peptide feature are averaged to generate a list of “implied-identified peptides”. Robust fit linear regression is performed for each dataset using the “implied-identified peptides” as predictors, and the measured identified peptides as response. The resulting intercept is taken as the mean hyperparameter in the shift prior distribution (*a*_*d*_). Similarly, the coefficient is taken as the mean hyperparameter in the scale prior distribution (*e*_*d*_). The variance parameters on the shift and scale priors (*b*_*d*_ and *f*_*d*_) are set tightly to the variance of the regression estimate. This allows an optimal solution to be reached as latent peptides are updated and added, while reducing shift and scale identifiability issues. Both the match covariance (*Σ*), and latent peptide covariance (*σ*) matrices are given conjugate inverse-Wishart priors as shown in Equations 6 and 8. The residuals of the shifted and scaled identified peptide measurements, and their respective “implied-identified peptides” are used set hyperparameters. The degrees of freedom parameters (*h* and *t*) are set to the number of identified matches minus one, and the inverse-scale matrix is set to the sum of squared residuals. The mean of each latent peptide (*μ*_*j*_) is given a conjugate normal prior with as shown in Equation 7. The prior mean (*λ*) is set to the empirical mean of all measured peptide features in all datasets, and the prior covariance (*r*) is set to the sum of squared differences between this empirical mean and all measured data. We express the likelihood of *x*_*d*,*i*_ as follows:

(9) $$\begin{array}{ll} \;P(x_{d,i}| & \eta ,\beta ,\Sigma ,z_{1}\mathrm{...}z_{J},c_{d,i})=\text{Normal}(x_{d,i}|\eta_{d}+z_{c_{d,i}}\beta_{d},\Sigma )\; \end{array}$$

Where *z*_1_...*z*_*J*_ indicates all existing latent peptides. We may also integrate out $z_{c_{d,i}}$ and re-express the likelihood as:

(10) $$\begin{array}{ll} \;P(x_{d,i}| & \eta ,\beta ,\Sigma ,\mu_{1}\mathrm{...}\mu_{J},\sigma ,c_{d,i})=\text{Normal}(x_{d,i}|A_{c_{d,i}},B_{d})\; \end{array}$$

() $$\begin{array}{lll} A_{c_{d,i}} & =\eta_{d}+\mu_{c_{d,i}}\beta_{d}\; & \;\\ B_{d} & =\beta_{d}^{T}\sigma \beta_{d}+\Sigma \; & \; \end{array}$$

The prior probability of an observation, *x*_*d*,*i*_, being assigned to latent peptide component *j* given all other assignment indicators, *c*_−(*d*,*i*)_, is given in Equation 11. The notation *c*_−(*d*,*i*)_ refers to all component indicators from all features in all datasets, except *d*,*i*. Similarly the prior probability of observation *x*_*d*,*i*_ being assigned to a new latent peptide component is shown in Equation 12.

(11) $$\begin{array}{ll} & \;p(c_{d,i}=j|c_{-(d,i)},\alpha )=\frac{n_{-(d,i),j}}{N-1+\alpha }\times I(!\exists i,c_{d,-i}=c_{d,i})\; \end{array}$$

(12) $$\begin{array}{ll} & p(c_{d,i}\neq c_{-(d,i)}|c_{-(d,i)},\alpha )=\frac{\alpha }{N-1+\alpha }\; \end{array}$$

Let *α* be the DPGMM concentration parameter, *N* the total number of observed peptide features across all samples, and *n*_−(*d*,*i*)_ the number of observed peptide features other than *x*_*d*,*i*_ assigned to latent peptide *j*. A constraint is imposed such that only one measurement per dataset may be assigned to a given latent peptide feature. The concentration parameter of the Dirichlet Process (*α*) is set to the number of peptide feature observations across all samples being aligned. Latent peptide feature assignments are updated from their full conditional posterior distributions, as shown in Equation 13.

(13) $$\begin{array}{lll} \;P(c_{d,i}=j|-)\; & \propto \frac{\alpha }{N-1+\alpha }\times \int P(x_{d,i}|-)\; & \;\\ & \;\times P(\mu_{j}|\lambda ,r)d\mu_{j}\; & \;\\ & \;+\;\underset{j}{\sum }\;\frac{n_{-(d,i),j}}{N-1+\alpha }\text{Normal}(x_{d,i}\;|\;A_{c_{d,i}},B_{d})\; & \;\\ & \;\times I(!\exists i,c_{d,-i}=c_{d,i})\; \end{array}$$

**Figure 4:**
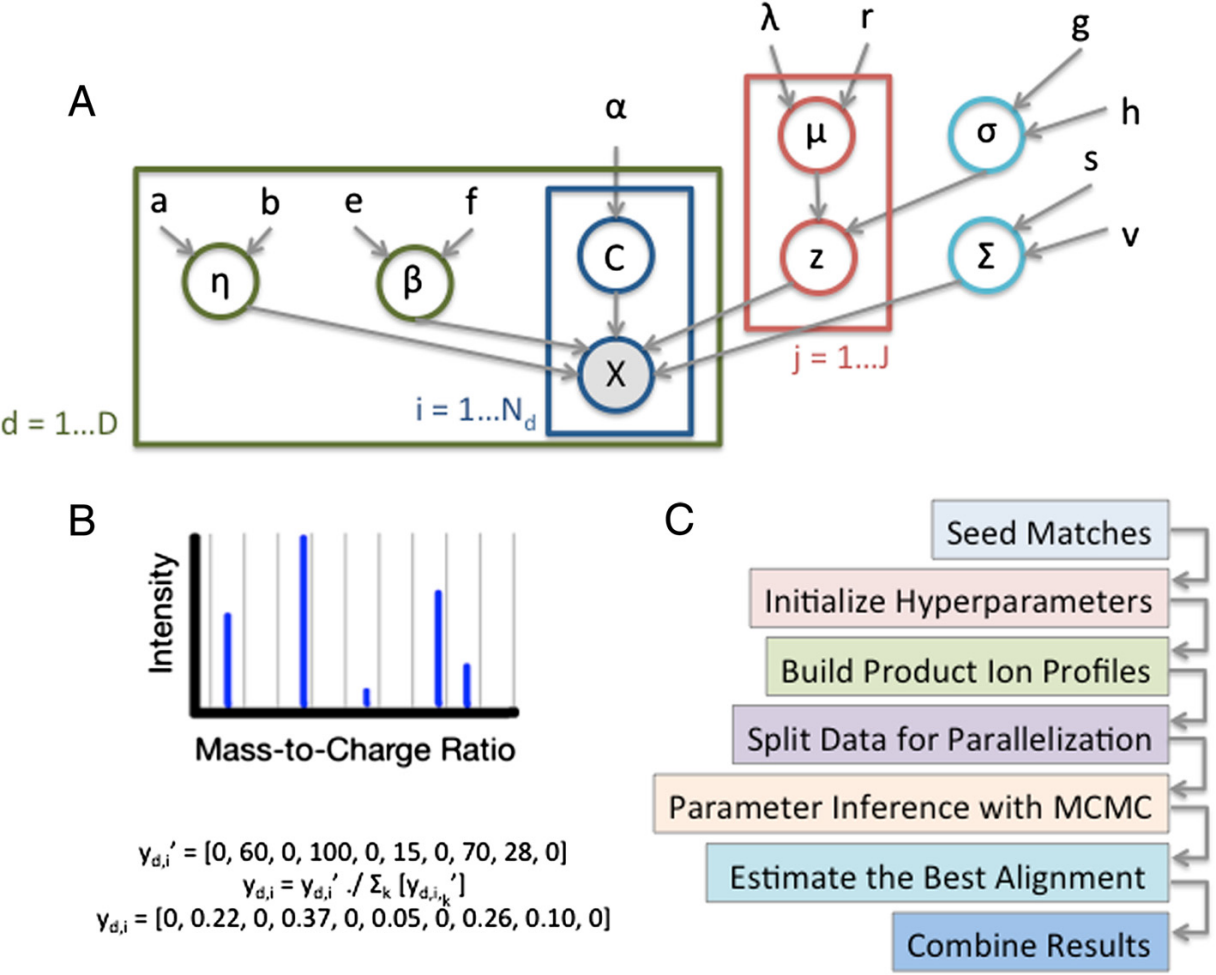
**Alignment algorithm steps and principles.** Panel **A** shows a Plate Diagram of the DPGMM Peptide-Level Model. Panel **B** illustrates the computation of a product ion profile with a small example of profile size *K*=10. Panel **C** gives an overview of the alignment algorithm steps.

The integral above is tractable due to the shared covariance across latent peptide components. Further details and full conditional distributions are available in Additional file 1. Panel A of Figure 4 shows a plate diagram of the peptide level alignment model.

#### Production model extension

**Figure 5:**
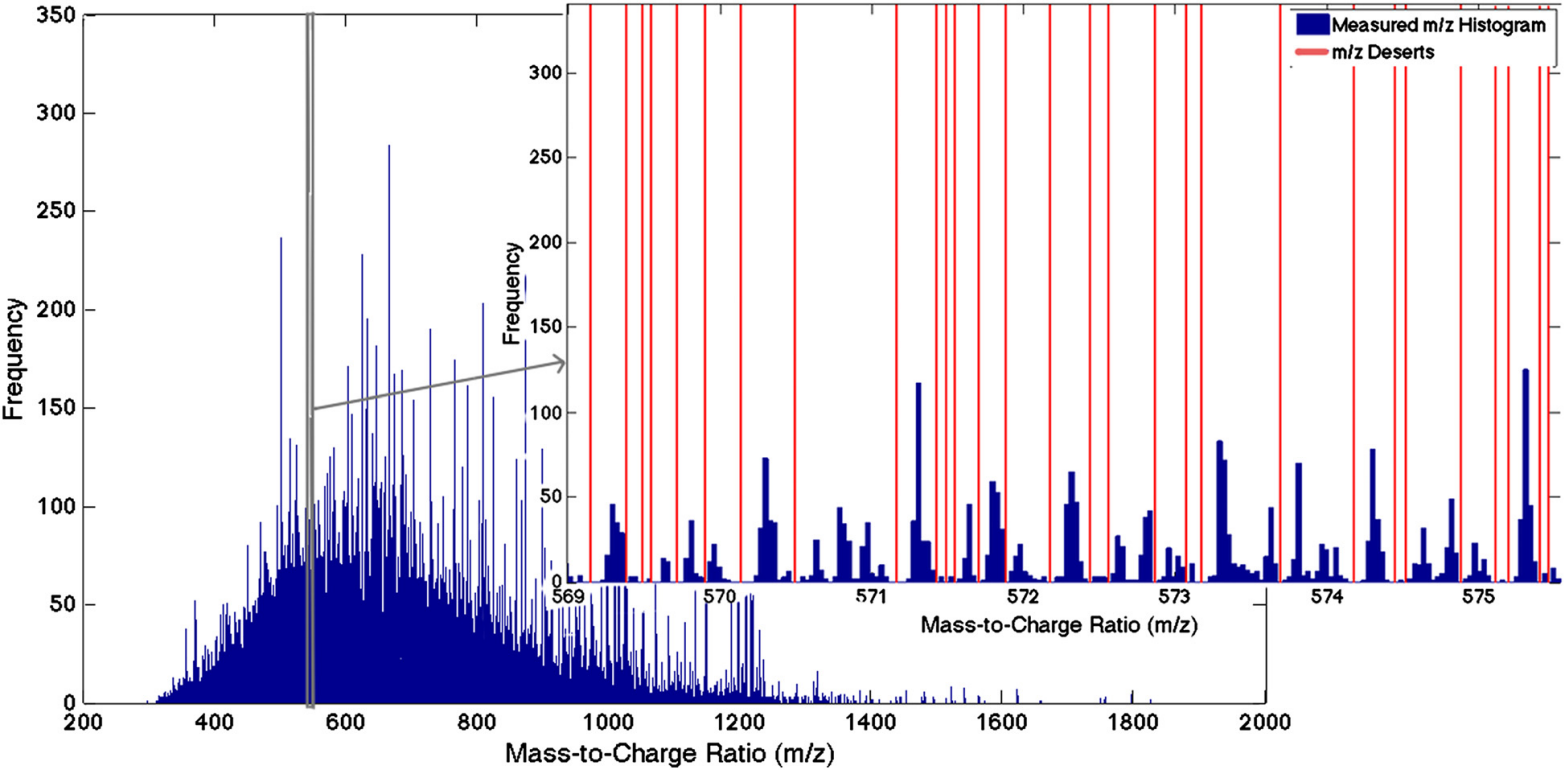
**Mass-to-charge ratio deserts.** Figure 5 illustrates the mass-to-charge ratio deserts. There are subsets of the mass-to-charge ratio dimension not occupied by any peptide features. We term these subsets mass-to-charge ratio “deserts” and utilize them to split the data for parallelization. The deserts are empirically determined using all datasets in the alignment. Shown is a histogram of measured m/z values for *E. coli* lysate data, the empirical m/z deserts are indicated with vertical red lines.

To incorporate production data, we select up to the 50 most intense productions for each peptide feature measurement, *x*_*d*,*i*_. We then generate a *K*-dimensional production intensity profile for each *x*_*d*,*i*_. Each position, $y_{d,i_{k}}$, in the product ion intensity profile, *y*_*d*,*i*_, is computed as:

(14) $$\begin{array}{l} y_{d,i_{k}}=\frac{\underset{p}{\sum }\Omega_{p}\times I(M_{p}\leq B_{k})}{\underset{p}{\sum }\Omega_{p}} \end{array}$$

where *k*=1…*K*, *p*=1…50, *Ω* is a 50-dimensional vector of intensities, *M* is a 50-dimensional vector of product ion mass-to-charge ratios, and *B* is a K-dimensional vector of product ion profile mass-to-charge ratio bin upper limits. All values in *y*_*d*,*i*_ sum to one. The mass-to-charge ratio ranges, or bins (*B*_*k*_), are determined at the initialization of the alignment, such that bin boundaries fall on mass-to-charge ratio “deserts”. See the Parellelization section and Figure 5 for a detailed description of bin boundary determination. In the experiments described here, we set K = 250 to ensure most product ions would be assigned to their own bin in the product ion profile, and to avoid additional computational complexity. Each existing latent peptide feature is given a K-dimensional product ion profile (*w*_*j*_ for latent peptide feature *z*_*j*_). To assess the similarity of a measured product ion profile, *y*_*d*,*i*_, and a latent production profile, $w_{c_{d,i}}$, we introduce a similarity score, *ψ*, which is computed as the sum of squared differences of the two product ion intensity profiles, and is assumed to have an exponential distribution to encourage distances close to zero, as shown in Equations 15 and 16.

(15) $$\begin{array}{ll} & \psi_{d,i}={\left(y_{\text{di}}-w_{c_{d,i}}\right)}^{T}(\;y_{d,i}-w_{c_{d,i}})\; \end{array}$$

(16) $$\begin{array}{ll} & \psi_{d,i}\;\;∼\text{Exponential}(\gamma )\; \end{array}$$

We assign a conjugate gamma prior to the rate parameter, as shown in Equation 17. The hyperparameters for profile scores are set to one.

(17) $$\begin{array}{l} \gamma ∼\text{Gamma}(a_{0},b_{0}) \end{array}$$

At each iteration of the MCMC, the product ion profile, *w*_*j*_ of an existing latent peptide is updated empirically – the product ion profile is set to the average of the measured product profiles assigned to that latent peptide. The latent product ion profile, *w*_0_, of a new latent peptide (one that currently does not exist) is a blank profile - a uniform vector of size K with each element having value 1/K. Combining the product ion model with the peptide-level model, we have the following likelihood (Equation 18) and conditional posterior (Equation 19):

(18) $$\;\begin{array}{ll} P(x_{d,i},y_{d,i}|-) & =\text{Normal}(x_{d,i}|A_{c_{d,i}},B_{d})\\ & \;\times \text{Exponential}(\psi_{d,i}|\gamma ) \end{array}$$

(19) $$\;\begin{array}{ll} P(c_{d,i}=j|-)\propto \frac{\alpha }{N-1+\alpha } & \times \int P(x_{d,i}|-)\\ & \times P(\mu_{j}|\lambda ,r)d\mu_{j}\\ & \times \text{Exponential}({\left(\;y_{\text{di}}-w_{0}\right)}^{T}\\ & \times (y_{d,i}-w_{0})|\gamma )\\ & +\underset{j}{\sum }\frac{n_{-(d,i),j}}{N-1+\alpha }\\ & \times \text{Normal}(x_{d,i}|A_{c_{d,i}},B_{d})\\ & \times I(!\exists i,c_{d,-i}=c_{d,i})\\ & \times \text{Exponential}(\psi_{d,i}|\gamma ) \end{array}$$

Further details and full conditional distributions are available in Additional file 1. We explored additional values of *K*, as well as implementations of different product ion models, the results and discussions of which can also be found in Additional file 1.

#### Model fitting

#### Posterior match probabilities

As our primary goal is obtaining a list of matches, we are only interested in maximum a posteriori (MAP) estimates of the parameters. We employ simulated annealing on all parameters after the initial burn-in period of the MCMC. In addition, with the exception of the latent peptide means, the model parameters are being updated with a large number of observations, and will have fairly tight posterior distributions. Our model assumes that the product ion match likelihoods are independent from the peptide-level match likelihoods. New peptide match indicators are sampled from the full conditionals for each measured peptide in a random order. We sample match indicators in accordance with “Algorithm 2” for sampling mixture component indicators in DPGMM from Neal, 2000. We impose a restriction on match assignment such that only one measured peptide per dataset may be assigned to a given latent peptide. All other parameters are sampled from their full conditional distributions. After obtaining MAP estimates for all parameters, we then iteratively re-sample only the component assignment indicators, keeping track of how often each measurement is assigned to each latent peptide. These assignment proportions are used to make final matches.

#### Estimating the best alignment

Utilizing the assignment proportions from the final “assignment-only” MCMC iterations, we use a greedy algorithm to determine the final alignment. The best match (latent peptide-measurement pair) across the entire alignment is selected, and then the assignment proportions for measurements in each of the remaining datasets are examined for the current latent peptide. For each dataset, the measurement with the maximum assignment proportion is selected. All remaining match probabilities for the assigned measurements and the current latent peptide are set to zero. This process is repeated until no non-zero assignment proportions remain. These assignment probabilities represent the probability that a given measurement arises from a certain latent peptide. To compute the match probability of two measurements from two datasets, we compute the probability that both measurements are assigned to the same latent peptide – the product of the two individual latent peptide assignment probabilities. Users may utilize these match probabilities to examine matches of varying confidence. An illustration of the algorithm steps is shown in Panel C of Figure 4.

#### Parallelization

In order to make alignment of large datasets tractable, we split the datasets being aligned in the mass-to-charge ratio dimension, and perform separate alignments of each split in parallel. The boundaries of these splits fall only on mass-to-charge ratio “deserts”, which are empirically determined using all datasets being aligned. There exist gaps - also called “forbidden zones” in the mass distribution of all possible tryptic peptides [39]. These gaps have been utilized to improve peak de-noising techniques [40]. We calculate these gaps empirically based on the data in question, and utilize them to split the data for alignment. Such mass-to-charge ratio deserts are shown in Figure 5. When determining these mass-to-charge ratio deserts, we utilize the given matches to determine an approximate shift, scale, and match standard deviation. We then obtain the number of measured peptides in each mass-to-charge ratio bin the size of match standard deviation, and split the datasets at mass-to-charge ratio deserts defined as stretches of five or more empty bins. This ensures that any measured peptide features with the potential for being aligned to the same latent peptide feature (any measurements that should match one another) will be in the same alignment split. The hyperparameters set in the model initialization are shared across all alignment splits.

The boundaries of the product ion profiles are determined in a similar way. Utilizing the product ion annotations of the given matches, we obtain a mass-to-charge ratio match standard deviation of product ions. We then obtain the number of measured product ions in each mass-to-charge ratio bin the size of the match standard deviation, and set the product ion profile boundaries on mass-to-charge ratio deserts defined as stretches of three or more empty bins. The size of the product ion profile boundaries is as close to 1/K of the spanning product ion mass-to-charge ratio range as possible, given the boundaries are set within mass-to-charge ratio deserts.

### Data

All data used in this analysis was obtained under MS^E^ and/or HDMS^E^ conditions (SYNAPT HDMS G2, Waters), and subject to Waters ProteinLynx Global SERVER (PLGS) processing. We utilize peptide features that have already been subject to peak detection, de-isotoping, charge state determination, and tentative identification (although not all identifications are utilized for alignment). All samples were separated by 1D nanoscale capillary ultraperformance Liquid Chromatography in a 90-minute gradient using a 5–40% acetonitrile/water (0.1% formic acid in each).

Three HCV cohorts were utilized in the alignment of serum samples from HCV patients to urine samples from OA patients. The first cohort included 47 patients ages 5 to 18 years from a clinical trial for HCV treatment [41]. The two additional HCV cohorts (n = 41,55) were selected from the Duke Hepatology Clinical Research (DHCR) database [42]. The pediatric clinical trial study was approved by the institutional review boards of the participating sites. Written informed consent was provided by all parents or guardians, and written assent was provided by all participants over 12 years of age. All patients present in the DHCR database cohorts, as well as all OA patients, provided written informed consent, and all study procedures were approved by the Duke University Institutional Review Board.

### Analysis

All alignments were performed using Matlab on the Duke Shared Computing Resource, a cluster of Intel x86 compute notes running Linux. Each alignment was partitioned into a maximum of 250 splits, and each alignment partition was run on a single node with at least 8GB of memory. Our method does require considerable computation time - approximately 2 to 6 hours for the *E. coli* Lysate and HCV-OA alignments not utilizing product ions, and approximately 20–24 hours for the *E. coli* Lysate alignments utilizing product ions. Times vary by the number and size of datasets being aligned.

## Abbreviations

AMT: Accurate mass and time; DDA: Data depenent acquisition; DIA: Data independent acquisition; DPGMM: Dirichlet process Gaussian mixture model; HCV: Hepatitis-C virus; HDMS^E^: Waters Corporation “high-low switching” fragmentation mass spectrometry coupled with ion mobility separation; IM: Ion mobility; MAP: Maximum a posteriori; MCMC: Markov chain Monte Carlo; MS^E^: Waters Corporation “high-low switching” fragmentation mass spectrometry; MS/MS: Tandem mass spectrometry; MZ-IM: Mass-to-charge ratio and ion mobility drift time alignment; MZ-RT: Mass-to-charge ratio and retention time alignment; MZ-RT-IM: Mass-to-charge ratio, retention time, and ion mobility drift time alignment; MZ-RT-IM-HE: Mass-to-charge ratio, retention time, ion mobility drift time, and product ion data alignment; PLGS: ProteinLynx Global SERVER, The Waters informatics platform for processing proteomics data; RTCC: Retention time calibration curve.

## Competing interests

AMB, RH, and JL have no competing interests to declare. SJG is employed by Waters, the creators of MS^E^ and HDMS^E^. JWT, EJS, and MAM are a part of the Duke Proteomics Core Facility, a Waters Center of Innovation. JWT, EJS, and MAM receive no direct financial support from Waters.

## Authors’ contributions

JL, MAM, and AMB designed the method. RH, JWT, SJG, and EJS provided insight for essential modifications to method design. JWT conceived the test studies. VBK provided access to data and clinical insight for identification carryover results. AMB implemented the method, carried out the analyses, and drafted the manuscript. EJS ran all experiments to obtain the data. All authors read and approved the final manuscript.

## Supplementary Material

Additional file 1 **Software Documentation and Supplemental Results.** Additional file 1 contains information on how to use our Matlab code to perform alignments, the full conditional distributions to update each parameter in the model, an brief exploratory analysis of different product ion models, and supplemental results and figures. The supplemental results include mismatches and recall rates using different PLGS peptide score thresholds for the *E. coli* lysate analysis, the inverse decoy experiment (aligning technical replicates of *E. coli* lysate with a human plasma decoy), and information about the identification carryover analysis (results for this analysis are in Additional file 2). Click here for file

Additional file 2 **Supplemental Tables.** Additional file 2 is an excel spreadsheet containing all supplemental tables. Additional file 2: Tables S1 and S2 show the p-values comparing recall rates and mismatches, respectively, across the different alignments. Additional file 2: Table S3 shows the inferred Hepatitis-C proteins from the identification carryover analysis. Additional file 2: Table S4 shows the GATHER Gene Ontology enrichment results for the inferred proteins, and Additional file 2: Table S5 shows the DAVID Biological Process Gene Ontology enrichment results for the inferred proteins. Additional file 2: Table S6 shows the GATHER chomosome location enrichment results for the inferred Hepatitis-C proteins. Click here for file

Additional file 3 **Sample Configuration File.** Additional file 3 is a sample configuration file to set up an alignment. See Additional file 1 for more details. Click here for file

Additional file 4 **Sample Queue Submission File.** Additional file 4 is a sample SGE queue submission file to start an alignment. See Additional file 1 for more details. Click here for file

Additional file 5 **Alignment Model Matlab Code.** Additional file 5 contains the Matlab code for our alignment method. See Additional file 1 for more details. Click here for file

## References

[B1] Gillet LC Navarro P Tate S Röst H Selevsek N Reiter L Bonner R Aebersold R **Targeted data extraction of the MS/MS spectra generated by data-independent acquisition: a new concept for consistent and accurate proteome analysis** Mol Cell Proteomic 2012 14 6 O111.016717 10.1074/mcp.O111.016717 PMC3433915 22261725

[B2] Geromanos SJ Vissers JP Silva JC Dorschel CA Li GZ Gorenstein MV Bateman RH Langridge JI **The detection, correlation, and comparison of peptide precursor and product ions from data independent LC-MS with data dependent LC-MS/MS** Proteomics 2009 14 1683 1695 10.1002/pmic.200800562 19294628

[B3] Dowsey AW English JA Lisacek F Morris JS Yang GZ Dunn MJ **Image analysis tools and emerging algorithms for expression proteomics** Proteomics 2010 14 23 4226 4257 10.1002/pmic.200900635 21046614 PMC3257807

[B4] Zhang JQ Gonzalez E Hestilow T Haskins W Huang YF **Review of peak detection algorithms in liquid-chromatography-mass spectrometry** Curr Genomics 2009 14 6 388 401 10.2174/138920209789177638 20190954 PMC2766790

[B5] Listgarten J Emili A **Statistical and computational methods for comparative proteomic profiling using liquid chromatography-tandem mass spectrometry** Mol Cell Proteomic 2005 14 4 419 434 10.1074/mcp.R500005-MCP200 15741312

[B6] Bell AW Deutsch EW Au CE Kearney RE Beavis R Sechi S Nilsson T Bergeron JJ **A HUPO test sample study reveals common problems in mass spectrometry-based proteomics** Nat Methods 2009 14 6 423 430 10.1038/nmeth.1333 19448641 PMC2785450

[B7] Jeffries N **Algorithms for alignment of mass spectrometry proteomic data** Bioinformatics 2005 14 14 3066 3073 10.1093/bioinformatics/bti482 15879456

[B8] Service RF **Proteomics. Proteomics ponders prime time** Science 2008 14 5897 1758 1761 10.1126/science.321.5897.1758 18818332

[B9] Prince JT Marcotte EM **Chromatographic alignment of ESI-LC-MS proteomics data sets by ordered bijective interpolated warping** Anal Chem 2006 14 17 6140 6152 10.1021/ac0605344 16944896

[B10] Vissers JPC Langridge JI Aerts JMFG **Analysis and quantification of diagnostic serum markers and protein signatures for Gaucher disease** Mol Cell Proteomic 2007 14 5 755 766 10.1074/mcp.M600303-MCP200 17293593

[B11] Silva JC Denny R Dorschel C Gorenstein MV Li GZ Richardson K Wall D Geromanos SJ **Simultaneous qualitative and quantitative analysis of the Escherichia coli proteome: a sweet tale** Mol Cell Proteomic 2006 14 4 589 607 10.1074/mcp.M500321-MCP200 16399765

[B12] Li XJ Yi EC Kemp CJ Zhang H Aebersold R **A software suite for the generation and comparison of peptide arrays from sets of data collected by liquid chromatography-mass spectrometry** Mol Cell Proteomic 2005 14 9 1328 1340 10.1074/mcp.M500141-MCP200 16048906

[B13] Silva JC Denny R Dorschel CA Gorenstein M Kass IJ Li GZ McKenna T Nold MJ Richardson K Young P Geromanos S **Quantitative proteomic analysis by accurate mass retention time pairs** Anal Chem 2005 14 7 2187 200 10.1021/ac048455k 15801753

[B14] Zhang XA Asara JM Adamec J Ouzzani M Elmagarmid AK **Data pre-processing in liquid chromatography-mass spectrometry-based proteomics** Bioinformatics 2005 14 21 4054 4059 10.1093/bioinformatics/bti660 16150809

[B15] Katajamaa M Miettinen J Oresic M **MZmine: toolbox for processing and visualization of mass spectrometry based molecular profile data** Bioinformatics 2006 14 5 634 636 10.1093/bioinformatics/btk039 16403790

[B16] Bellew M Coram M Fitzgibbon M Igra M Randolph T Wang P May D Eng J Fang RH Lin CW Chen JZ Goodlett D Whiteaker J Paulovich A McIntosh M **A suite of algorithms for the comprehensive analysis of complex protein mixtures using high-resolution LC-MS** Bioinformatics 2006 14 15 1902 1909 10.1093/bioinformatics/btl276 16766559

[B17] Smith CA Want EJ O’Maille G Abagyan R Siuzdak G **XCMS: Processing mass spectrometry data for metabolite profiling using Nonlinear peak alignment, matching, and identification** Anal Chem 2006 14 3 779 787 10.1021/ac051437y 16448051

[B18] Wang P Tang H Fitzgibbon MP McIntosh M Coram M Zhang H Yi E Aebersold R **A statistical method for chromatographic alignment of LC-MS data** Biostatistics 2007 14 2 357 367 10.1093/biostatistics/kxl015 16880200

[B19] Lange E Gropl C Schulz-Trieglaff O Leinenbach A Huber C Reinert K **A geometric approach for the alignment of liquid chromatography-mass spectrometry data** Bioinformatics 2007 14 13 i273 i281 10.1093/bioinformatics/btm209 17646306

[B20] Sturm M Bertsch A Gropl C Hildebrandt A Hussong R Lange E Pfeifer N Schulz-Trieglaff O Zerck A Reinert K Kohlbacher O **OpenMS - an open-source software framework for mass spectrometry** BMC Bioinformatics 2008 14 163 10.1186/1471-2105-9-163 18366760 PMC2311306

[B21] Yu T Park Y Johnson JM Jones DP **apLCMS–adaptive processing of high-resolution LC/MS data** Bioinformatics 2009 14 15 1930 1936 10.1093/bioinformatics/btp291 19414529 PMC2712336

[B22] Pluskal T Castillo S Villar-Briones A Oresic M **MZmine 2: modular framework for processing, visualizing, and analyzing mass spectrometry-based molecular profile data** BMC Bioinformatics 2010 14 395 10.1186/1471-2105-11-395 20650010 PMC2918584

[B23] Jaffe JD Mani DR Leptos KC Church GM Gillette MA Carr SA **PEPPeR, a platform for experimental proteomic pattern recognition** Mol Cell Proteomic 2006 14 10 1927 1941 10.1074/mcp.M600222-MCP200 PMC2649820 16857664

[B24] Fischer B Grossmann J Roth V Gruissem W Baginsky S Buhmann JM **Semi-supervised LC/MS alignment for differential proteomics** Bioinformatics 2006 14 14 e132 e140 10.1093/bioinformatics/btl219 16873463

[B25] Tang Z Zhang L Cheema AK Ressom HW **A new method for alignment of LC-MALDI-TOF data** Proteome Sci 2011 14 Suppl 1 S10 10.1186/1477-5956-9-S1-S10 22166061 PMC3289071

[B26] Zhang Z **Retention time alignment of LC/MS data by a divide-and-conquer algorithm** J Am Soc Mass Spectrom 2012 14 4 764 772 10.1007/s13361-011-0334-2 22298290

[B27] Mueller LN Rinner O Schmidt A Letarte S Bodenmiller B Brusniak MY Vitek O Aebersold R Muller M **SuperHirn - a novel tool for high resolution LC-MS-based peptide/protein profiling** Proteomics 2007 14 19 3470 3480 10.1002/pmic.200700057 17726677

[B28] Nielsen NPV Carstensen JM Smedsgaard J **Aligning of single and multiple wavelength chromatographic profiles for chemometric data analysis using correlation optimised warping** J Chromatogr A 1998 14 1–2 17 35

[B29] Prakash A Mallick P Whiteaker J Zhang H Paulovich A Flory M Lee H Aebersold R Schwikowski B **Signal maps for mass spectrometry-based comparative proteomics** Mol Cell Proteomic 2006 14 3 423 432 10.1074/mcp.M500133-MCP200 16269421

[B30] Lange E Tautenhahn R Neumann S Gropl C **Critical assessment of alignment procedures for LC-MS proteomics and metabolomics measurements** BMC Bioinformatics 2008 14 375 10.1186/1471-2105-9-375 18793413 PMC2570366

[B31] West M **Mixture-models, Monte-Carlo, Bayesian updating and dynamic-models** Comput Sci Stat 1992 14 325 333

[B32] Rasmussen CE **The infinite Gaussian mixture model** Adv Neural Inf Process Syst 12 2000 14 554 560

[B33] Reich M Liefeld T Gould J Lerner J Tamayo P Mesirov JP **GenePattern 2.0** Nat Genet 2006 14 5 500 501 10.1038/ng0506-500 16642009

[B34] Li GZ Vissers JP Silva JC Golick D Gorenstein MV Geromanos SJ **Database searching and accounting of multiplexed precursor and product ion spectra from the data independent analysis of simple and complex peptide mixtures** Proteomics 2009 14 1696 1719 10.1002/pmic.200800564 19294629

[B35] Wilkins MR Williams KL **Cross-species protein identification using amino acid composition, peptide mass fingerprinting, isoelectric point and molecular mass: A theoretical evaluation** J Theor Biol 1997 14 7 15 10.1006/jtbi.1996.0346 9176634

[B36] Chang JT Nevins JR **GATHER: a systems approach to interpreting genomic signatures** Bioinformatics 2006 14 23 2926 2933 10.1093/bioinformatics/btl483 17000751

[B37] Dennis JG Sherman BT Hosack DA Yang J Gao W Lane HC Lempicki RA **DAVID: Database for Annotation, Visualization, and Integrated Discovery** Genome Biol 2003 14 5 P3 10.1186/gb-2003-4-5-p3 12734009 PMC193660

[B38] Lam H **Building and searching tandem mass spectral libraries for peptide identification** Mol Cell Proteomic 2011 14 12 R111.008565 10.1074/mcp.R111.008565 PMC3237092 21900153

[B39] Nefedov AV Mitra I Brasier AR Sadygov RG **Examining Troughs in the mass distribution of all theoretically possible tryptic peptides** J Proteome Res 2011 14 4150 4157 10.1021/pr2003177 21780838 PMC3184890

[B40] Mitra I Nefedov AV Brasier AR Sadygov RG **Improved mass defect model for theoretical tryptic peptides** Anal Chem 2012 14 3026 3032 10.1021/ac203255e 22401145 PMC3312599

[B41] Schwarz KB Gonzalez-Peralta RP Murray KF Molleston JP Haber BA Jonas MM Rosenthal P Mohan P Balistreri WF Narkewicz MR Smith L Lobritto SJ Rossi S Valsamakis A Goodman Z Robuck PR Barton BA Peds-C Clinical Research Network **The combination of ribavirin and peginterferon is superior to peginterferon and placebo for children and adolescents with chronic hepatitis C** Gastroenterology 2011 14 2 e1 10.1053/j.gastro.2010.03.070 21036173 PMC3042126

[B42] Patel K Lucas JE Thompson JW Dubois LG Tillmann HL Thompson AJ Uzarski D Califf RM Moseley MA Ginsburg GS McHutchison JG McCarthy JJ MURDOCK Horizon 1 Study Team **High predictive accuracy of an unbiased proteomic profile for sustained virologic response in chronic hepatitis C patients** Hepatology 2011 14 6 1809 1818 10.1002/hep.24284 21381069

