# Supplementary material for: A flexible statistical model for alignment of label-free proteomics data – incorporating ion mobility and product ion information
Source: BMC Bioinformatics. 2013 Dec 16;14:364. doi: 10.1186/1471-2105-14-364 (PMC3878627; doi:10.1186/1471-2105-14-364)
Supplement: Additional file 1 — Software Documentation and Supplemental Results. Additional file 1 contains information on how to use our Matlab code to perform alignments, the full conditional distributions to update each parameter in the model, an brief exploratory analysis of different product ion models, and supplemental results and figures. The supplemental results include mismatches and recall rates using different PLGS peptide score thresholds for the E. coli lysate analysis, the inverse decoy experiment (aligning technical replicates of E. coli lysate with a human plasma decoy), and information about the identification carryover analysis (results for this analysis are in Additional file 2). [file 1471-2105-14-364-S1.pdf]

1 Software and Data Formatting

1.1 Data Processing Script

We have written a data-processing script to read Waters 'spectrum.xml' and 'finalfrag.csv' files into a Matlab data frame for subsequent alignment with our software. The script and accompanying functions are in the software archive. The "prepareDatasets" function takes a variety of input file formats, pre-processes intensities, and creates a data structure for the alignment software. It can be called as follows from the Matlab command window: datasets = prepareDatasets(file_names, options, data_range, peptide_list, sample_list) The variable "file_names" should be a cell array of strings. Each row should be a dataset (or single sample), with each column containing a different file for that dataset. This function is designed to handle 4 different input types:

1. 2 files listed in this order: spectrum.xml, finalfrag.csv (Water's pipeline output)
2. 1 file: spectrum.xml (Water's pipeline output)
3. 1 file: finalfrag.csv (Water's pipeline output)
4. 1 file: data.mat (un-processed matlab data frame).

The variable "options" should be a 7x1 or 1x7 vector of numbers indicating a user's selections for the following options (in order): set very low intensity values to missing (0 for off, or a raw intensity lower limit), impute the missing intensity values (0 for off and 1 for on), combine replicates (0 for off and 1 for on), the maximum number of product ions to store per peptide (most intense stored first), a peptide annotation quality limit (1 for "Pass 1", 2 for "Pass 1" and "Pass 2"), an option for log-transforming the intensities (0 for off and 1 for on), and an option for mean-centering the intensities for each sample. This input vector is optional, but must be included if any of the subsequent input parameters are used. To use the default values, the user may simply specify an empty vector [] in its place. The variable "data_range" should be a 3x2 matrix of numbers indicating the minimum and maximum (inclusive) data ranges for the mass-to-charge ratio, retention time, and drift time dimensions, respectively. The first column should contain the minimum values and the second, the maximum values. This input matrix is optional, but must be included if any of the subsequent input parameters are used. To use the entire data range, the user may simply specify an empty matrix [] in it's place. To only specify a minimum OR a maximum, or only limit certain dimensions, use NaNs in the unlimited slots. The variable "peptide_list" should be a cell array of peptide ids to include in the prepared data. Each row should correspond to a dataset, and each column a peptide id. This input

cell array is optional, but must be included if any of the subsequent input parameters are used. To use all peptides, the user may simply specify an empty vector [] in it's place. The variable "sample_list" should be a cell array of sample ids to include in the prepared data. Each row should correspond to a dataset, and each column a sample id. With single-sample files, the corresponding sample ID in this list will be used in the resulting data frame. With aggregate data, the sample IDs in this list will be used to select specific samples to include in the data frame. This input cell array is optional. To use all samples for aggregate data, the user may simply specify an empty vector [] in its place.

1.2 Data Frame Format

The output, "datasets", will be a list of data frames containing the following fields:

sids Sample IDs

pids Peptide IDs

data Mass-to-Charge Ratio, Retention Time, and Ion Mobility

xpr Intensities (Rows-Peptides, Columns-Samples)

key Sample Key Information

keyHead Key Header

anno Peptide Annotation Information

annoHead Annotation Header

e Peptide Charge State

pep Modified Peptide Sequence (or "-" if missing)

pCode Protein Code (or "-" if missing)

productmz Product Ion Mass-to-Charge Ratios

productints Product Ion Intensities (Raw)

productanno Product Ion Annotations (y1, y2, b7, etc.)

The prepareDatasets function can be easily adapted to incorporate any additional separation dimensions similar to ion mobility and liquid chromatography by appending additional columns into the data field. If you wish to use your own files in other formats, you may adapt the prepareDatasets function, or write your own function to generate a data frame as specified above.

1.3 Alignment Setup

To run your own alignment, you should set up a config file much like "sampleconfig.txt". In your config file, there should be the following specifications in order:

%DESCRIPTION: On the line following this tag, include a description of your alignment. This is for your record-keeping purposes only and does not affect the alignment.

%FILENAMES: On the lines following this tag, you should include the file(s) for your datasets being aligned. These should be one dataset (or sample) per line, and may be specified 4 different ways:

- Two files separated by commas and listed in this order: spectrum.xml,finalfrag.csv (Water's pipeline output)
- One file: spectrum.xml (Water's pipeline output)
- One file: finalfrag.csv (Water's pipeline output)
- One file: data.mat (file containing a matlab data frame in the format specified above)

%DIMNAMES: On the line following this tag, you should list the peptide-level separation dimensions you wish to use in your alignment, separated by commas. If you are using HDMSE data and want to use all three dimensions, the line should read: "Monoisotopic m/z,Peak Centroid Time,Ion Mobility"

%NGIVEN: This is the number of shared identifications that the model will "trust." These will remain matched at all iterations of the MCMC and be used as the seed matches to set hyperparameters. If you would like to trust all identifications in your data, specify "inf".

%MEASHE: The value on the line following this tag should be the maximum number of measured product ions to use per peptide (most intense used first). If you don't want to run a HE alignment, set this to 0.

%HEVECT: The value on the line following this tag should be the profile size K of the product ion profiles.

%ITERATIONS: There should be three value lines following this tag. The first is the number of burn-in iterations for the Gibb's Sampler, the second is the number of iterations post burn-in, and the third is the number of "assignment-only" iterations used to estimate match probabilities and the final alignment.

%NSPLIT: The value following this tag is the maximum number of "splits" to use to parallelize data alignment. If you are running your alignment on a cluster, each split will be set up as a separate job. If you are running your alignment locally, each split will be run consecutively after the previous split finishes.

%CODE: The line following this tag should contain the path of the directory containing the alignment software.

%OUT: The line following this tag should contain the path of the desired directory for results files.

%START_COMBINE: The line following this tag should contain either "START", or "COMBINE". The keyword "START" instructs the software to either start the alignment (if run locally) or set up the alignment (if run on a cluster. The keyword "COMBINE" instructs the software to combine the alignment splits – this should be run after all alignment partitions are finished and is only necessary if NSPLIT was set to a value greater than one.

%LOCAL_CLUSTER: The line following this tag should contain either "LOCAL", or "CLUSTER". The keyword "LOCAL" instructs the software to start the alignment on the local machine. The keyword "CLUSTER" sets up as many as NSPLIT qsub files and a submission bash script "submitall.sh" to be submitted to a SGE queue.

1.4 Running Your Alignment

If you are running your alignment locally using only 1 split, you can simply use the "Align" function provided in the software with your config file: "Align('config.txt')". If you are running your alignment locally using multiple splits, you should do the following:

1. Make sure the value line of the START_COMBINE tag in your config file says "START", and then run "Align('config.txt')" in your Matlab command window.

2. Modify the value line of the START_COMBINE tag in your config file to say "COMBINE".

3. After all alignment partitions have finished, run “Align(‘config.txt’)” in your Matlab command window once more.

If you are running your alignment in many partitions on a cluster, you should make a queue file for setting up your alignment like the file “sampleconfig.q”, and do the following:

1. Run “qsub sampleconfig.q” to set up your alignment.
2. Once the job in 1 finishes, run the submission script to start your alignment: “bash submitall.sh”.
3. Modify the value line of the START_COMBINE tag in your config file to say “COMBINE”.
4. After all alignment partitions have finished, run “qsub sampleconfig.q” once more to collect your alignment results.

1.5 Alignment Results Format

After your alignment, There will be 6 files containing various aspects of your alignment results. Each file is a matlab data frame and their contents are described in detail below.

split_datasets.mat This file contains your original datasets as they were split into mass-to-charge ratio positions

split_datasets A data structure of size NSPLIT x the number of datasets. Each element in the structure is a subset of the original data within a determined m/z range, having the format specified in section 1.1.2.

db.mat This file contains the new inferred dataset, combining the information from your aligned datasets into a single data frame.

db A data structure containing inferred peptide sequences, protein names, charge states, peptide intensities, and peptide ids for the latent peptides. The peptide intensities are collected from measured peptides aligned to the specific latent peptide, and are in the same order as the input datasets column-wise.

latentpeps A data structure containing the inferred monoisotopic m/z, retention time, and drift time, and the inferred product ion profiles of each latent peptide.

matchinfo.mat This file contains the original datasets and match information from the alignment.

datasets The original datasets in the format specified in section 1.1.2.

matches For each latent peptide (row), the indices of the assigned measured peptide from each dataset (column).

matched_pids For each latent peptide (row), the peptide ID (pids) of the assigned measured peptide from each dataset (column).

peps For each latent peptide (row), the peptide sequences of the assigned measured peptide from each dataset (column).

pcodes For each latent peptide (row), the protein name of the assigned measured peptide from each dataset (column)

accuracy.mat This file contains information about the number of matches, correct matches, and incorrect matches based on the identifications in your data. This is only relevant if the number of seed matches given to the model was fewer than the number of shared identifications.

nummatches The number of matches obtained between dataset i (dimension 1) and dataset j (dimension 2) at match probability cutoff k (dimension 3) or greater. Values of match probability cutoffs are 0.1, 0.2, 0.3, 0.4, 0.5, 0.6, 0.7, 0.8, 0.9, and 1.

case1 The number of correct matches obtained between dataset i (dimension 1) and dataset j (dimension 2), considering identifications of peptide score cutoff k (dimension 3) or greater, at match probability cutoff l (dimension 3) or greater. Values of peptide score cutoffs are 3, 4, 5, 6, 7, 8, 9 and 10, and values of match probability cutoffs are as listed above.

case2 The number of incorrect matches obtained between dataset i (dimension 1) and dataset j (dimension 2), considering identifications of peptide score cutoff k (dimension 3) or greater, at match probability cutoff l (dimension 3) or greater. Values of peptide score cutoffs are 3, 4, 5, 6, 7, 8, 9 and 10, and values of match probability cutoffs are as listed above.

case3 The number of matches of unknown accuracy, resulting from neither dataset having an identification, between dataset i (dimension 1) and dataset j (dimension 2), considering identifications of peptide score cutoff k (dimension 3) or greater, at match probability cutoff l (dimension 3) or greater. Values of peptide score cutoffs and values of match probability cutoffs are as listed above.

case4 The number of matches of unknown accuracy, resulting from one dataset having an identification that is not known to exist in the other, between dataset i (dimension 1) and dataset j (dimension 2),

considering identifications of peptide score cutoff k (dimension 3) or greater, at match probability cutoff l (dimension 3) or greater. Values of peptide score cutoffs and values of match probability cutoffs are as listed above.

case5 The number of incorrect matches, resulting from one dataset having an identification that exists elsewhere in the other, between dataset i (dimension 1) and dataset j (dimension 2), considering identifications of peptide score cutoff k (dimension 3) or greater, at match probability cutoff l (dimension 3) or greater. Values of peptide score cutoffs and values of match probability cutoffs are as listed above.

possible_case1 The number of possible correct matches (shared identifications) between dataset i (dimension 1) and dataset j (dimension 2), considering identifications of peptide score cutoff k (dimension 3) or greater. Values of peptide score cutoffs are as listed above.

model.mat This file contains information about the estimated model parameters.

mcmcparams A structure of size NSPLIT containing MAP estimates for the shift, scale, match covariance, and latent peptide covariance parameters for each partition of the alignment.

parameters A structure of size NSPLIT containing hyperparameters set by the seed matches, and alignment parameters as specified in the config file.

matchprobs For each latent peptide (row), the probability of assignment for the final matched measured peptide from each dataset (column).

matchinit A structure of size NSPLIT containing seed matches for each alignment split. For each latent peptide (row), the indices of the seed matched peptide from each dataset (column).

maxpepperds The maximum number of measured peptides from the datasets being aligned (the size of the largest dataset).

matchaverages.mat This file contains the results from the "assignment-only" iterations of the sampler.

matchaverages A sparse matrix containing the match probability of each measured peptide to each latent peptide, from the "assignment-only" iterations of the sampler.

2 Full Conditional Distributions

The full conditional posterior distributions used to update the shift, scale, residual covariance, latent peptides, latent peptide covariance, and match indicators are shown below.

Given D open-platform proteomics datasets/samples $X = x_{1,1}, ...x_{1,N_1}, ..., x_{2,1}, ...x_{2,N_2}, ..., x_{D,1}, ...x_{D,N_D}$ of N total peptide features, where $\sum_{d=1}^{D} N_d = N$ and x_{d,N_d} is k-dimensional, we fit the DPGMM, matching each measurement to one of J latent peptide features, $Z = z_1, ..., z_J$ where J is unbounded. A global, linear shift and scale of peptide-level data is simutaneously estimated to de-warp experimental variation. We first describe the model assuming for peptide-level, or precursor ion data, and then describe the extension to incorporate product ion information. The measurement $x_{d,i}$, assigned to latent peptide feature j is expected to be a shifted and scaled approximation of z_j, with dataset or sample-specific shift and scale parameters with Gaussian noise. The latent peptide features are modeled as components of the DPGMM - with z_j having mean μ_j and covariance σ, making the simplifying assumption that latent peptide feature precision is the shared across all latent peptide features. Conjugate priors are used for all model parameters.

$$
\begin{aligned}
x_{d,i} &= \eta_d + z_j \beta_d + \epsilon_{d,i} \qquad (1)\\
\epsilon_{d,i} &\sim Normal(0, \Sigma)\\
z_j &\sim Normal(\mu_j, \sigma)\\
\\
\eta_d &\sim Normal(a_d, b_d)\\
\beta_d &\sim Normal(e_d, f_d)\\
\mu_j &\sim Normal(\lambda, r)\\
\sigma &\sim iWishart(g, h)\\
\Sigma &\sim Inverse - Wishart(s, t)
\end{aligned}
$$

We adapt the Chinese Restaraunt Process formulation of the DPGMM, introducing indicator variables $c_{d,i}$ for latent peptide feature assignment, where $d = 1...D$, and $i = 1...N_d$ (one for each measurement) and $c_{d,i} \in \{1, 2, ...J\}$. Occupation numbers n_j for $j = 1...J$ are also introduced, where n_j is the number of $c = j$. We express the likelihood conditioned on the indicators.

$$
P(X \mid \eta, \beta, \Sigma, z_1...z_J, c_{1,1}...c_{D,N_D}) = \prod_{d=1}^{D} \prod_{i=1}^{N_d} Normal(x_{d,i} \mid \eta_d + z_{c_{d,i}} \beta_d, \Sigma) \qquad (2)
$$

We may also integrate out Z and re-express the likelihood as follows:

$$
P(X \mid \eta, \beta, \Sigma, \mu_1...\mu_J, \sigma, c_{1,1}...c_{D,N_D}) = \prod_{d=1}^{D} \prod_{i=1}^{N_d} Normal(x_{d,i} \mid \eta_d + \mu_{c_{d,i}} \beta_d, \beta_d^T \sigma \beta_d + \Sigma) \qquad (3)
$$

Given the above likelihood and prior distribution, we obtain the full conditional distribution of the global shift parameter, η_d, as follows:

$$\begin{aligned}
P(\eta_d \mid -) &\propto P(X \mid \eta_d, \beta_d, \Sigma, \mu_1 ... \mu_J, \sigma, c_{d,1} ... c_{d,N_d}) \times P(\eta_d \mid a_d, b_d) \\
&\propto \prod_{i=1}^{N_d} Normal(x_{d,i} \mid \eta_d + \mu_{c_{d,i}} \beta_d, \beta_d^T \sigma \beta_d + \Sigma) \times Normal(\eta_d \mid a_d, b_d) \\
&\propto (2\pi)^{-\frac{k}{2}} \mid b_d \mid^{-\frac{1}{2}} \; e^{-\frac{1}{2}(\eta_d - a_d)^T b_d^{-1} (\eta_d - a_d)} \times \prod_{i=1}^{N_d} (2\pi)^{-\frac{k}{2}} \mid (\beta_d^T \sigma \beta_d + \Sigma) \mid^{-\frac{1}{2}} \\
&\times exp \left(-\frac{1}{2} (x_{d,i} - \eta_d - \mu_{c_{d,i}} \beta_d)^T (\beta_d^T \sigma \beta_d + \Sigma)^{-1} (x_{d,i} - \eta_d - \mu_{c_{d,i}} \beta_d) \right) \\
&\propto Normal \left(W^{-1} \left[b_d^{-1} a_d + \left(\beta_d^T \sigma \beta_d + \Sigma \right)^{-1} \left(\sum_{i=1}^{N_d} x_{d,i} - \mu_{c_{d,i}} \beta_d \right) \right], W^{-1} \right) \\
W &= \left[b_d^{-1} + N_d (\beta_d^T \sigma \beta_d + \Sigma)^{-1} \right]
\end{aligned} \tag{4}$$

Similarly, we obtain the full conditional distribution of the global scale parameter, β_d, as follows:

$$\begin{aligned}
P(\beta_d \mid -) &\propto P(X \mid \eta_{d,k}, \beta_{d,k}, \Sigma_{k,k}, Z_{1,k} ... Z_{J,k}, c_{d,1} ... c_{d,N_d}) \times P(\beta_{d,k} \mid c_{d,k}, d_{d,k}) \\
&\propto \prod_{i=1}^{N_d} Normal \left(\frac{x_{d,i,k} - \eta_{d,k}}{Z_{c_{d,i},k}} - \beta_{d,k} \mid 0, \frac{\Sigma_{k,k}}{Z^2_{c_{d,i},k}} \right) \times Normal(\beta_{d,k} \mid c_{d,k}, d_{d,k}) \\
&\propto (2\pi)^{-\frac{1}{2}} d_{d,k}^{-\frac{1}{2}} e^{-\frac{1}{2}(\beta_{d,k} - c_{d,k})^2 d_{d,k}^{-1}} \times \prod_{i=1}^{N_d} (2\pi)^{-\frac{1}{2}} \frac{\Sigma_{k,k}}{Z^2_{c_{d,i},k}}^{-\frac{1}{2}} e^{-\frac{1}{2}(\frac{x_{d,i,k} - \eta_{d,k}}{Z_{c_{d,i},k}} - \beta_{d,k})^2 (\frac{\Sigma_{k,k}}{Z^2_{c_{d,i},k}})^{-1}} \\
&\propto Normal \left(\frac{\frac{c_{d,k}}{d_{d,k}} + \sum_{i=1}^{N_d} \frac{Z_{c_{d,i},k}}{\Sigma_{k,k}} (x_{d,i,k} - \eta_{d,k})}{\frac{1}{d_{d,k}} + \sum_{i=1}^{N_d} \frac{Z^2_{c_{d,i},k}}{\Sigma_{k,k}}}, \frac{1}{\frac{1}{d_{d,k}} + \sum_{i=1}^{N_d} \frac{Z^2_{c_{d,i},k}}{\Sigma_{k,k}}} \right)
\end{aligned} \tag{5}$$

We obtain the full conditional posterior distribution for the mean of each latent peptide as follows:

$$\begin{aligned}
P(\mu_j \mid -) &\propto P(X \mid \eta_d, \beta_d, \Sigma, \mu_j, \sigma, c_{1,1} ... c_{D,N_D}) \times p(\mu_j \mid \lambda, r) \\
&\propto \prod_{d,i:c_{d,i}=j} Normal((x_{d,i} - \eta_d) \beta_d^{-1} \mid \mu_{c_{d,i}}, \sigma + \beta_d^{-1} \Sigma \beta_d^{-1}) \times Normal(\mu_j \mid \lambda, r) \\
&\propto \prod_{d,i:c_{d,i}=j} (2\pi)^{-\frac{k}{2}} \mid \sigma + \beta_d^{-1} \Sigma \beta_d^{-1} \mid^{-\frac{1}{2}} \; e^{-\frac{1}{2}((x_{d,i} - \eta_d)\beta_d^{-1} - \mu_j)^T (\sigma + \beta_d^{-1} \Sigma \beta_d^{-1})^{-1} ((x_{d,i} - \eta_d)\beta_d^{-1} - \mu_j)} \\
&\times (2\pi)^{-\frac{k}{2}} \mid r \mid^{-\frac{1}{2}} \; e^{-\frac{1}{2}(\mu_j - \lambda)^T r^{-1} (\mu_j - \lambda)} \\
&\propto Normal \left(R^{-1} \left[r^{-1} \lambda + \sum_{d,i:c_{d,i}=j} (\sigma + \beta_d^{-1} \Sigma \beta_d^{-1})^{-1} (x_{d,i} - \eta_d) \beta_d^{-1} \right], R^{-1} \right) \\
R &= \left[r^{-1} + \sum_{d,i:c_{d,i}=j} (\sigma + \beta_d^{-1} \Sigma \beta_d^{-1})^{-1} \right]^{-1}
\end{aligned} \tag{6}$$

We derive the full conditional distribution for the residual covariance as follows:

$$\begin{aligned}
P(\Sigma \mid -) &\propto P(X \mid \eta, \beta, \Sigma, Z_1 ... Z_J, c_{1,1} ... c_{D,N_D}) \times P(\Sigma \mid s, \nu) \\
&\propto \prod_{d=1}^{D} \prod_{i=1}^{N_d} Normal(x_{d,i} - \eta_d - z_{c_{d,i}} \beta_d \mid 0, \Sigma) \times inverseWishart(\Sigma \mid s, \nu) \\
&\propto \prod_{d=1}^{D} \prod_{i=1}^{N_d} (2\pi)^{-\frac{k}{2}} \mid \Sigma \mid^{-\frac{1}{2}} e^{-\frac{1}{2}(x_{d,i} - \eta_d - z_{c_{d,i}} \beta_d)^T \Sigma^{-1} (x_{d,i} - \eta_d - z_{c_{d,i}} \beta_d)} \\
&\times \left[2^{\nu k/2} \pi^{\binom{k}{2}/2} \mid s \mid^{-\nu/2} \prod_{k=1}^{K} \Gamma((\nu + 1 - k)/2) \right]^{-1} \mid \Sigma \mid^{-(\nu+k+1)/2} e^{-tr(s\Sigma^{-1})/2} \\
&\propto iWishart([s + s_\theta]^{-1}, \nu + N) \\
s_\theta &= \sum_{d=1}^{D} \sum_{i=1}^{N_d} (x_{d,i} - \eta_d - z_{c_{d,i}} \beta_d)^T (x_{d,i} - \eta_d - z_{c_{d,i}} \beta_d)
\end{aligned} \tag{7}$$

The full conditional distribution for the shared latent peptide covariance is obtained as follows:

$$\begin{aligned}
P(\sigma \mid -) &\propto P(Z \mid \mu_1 ... \mu_j, \sigma) \times p(\sigma \mid g, h) \\
&\propto \prod_{j=1}^{J} Normal(z_j \mid \mu_j, \sigma) \times inverseWishart(\sigma \mid g, h) \\
&\propto \prod_{j=1}^{J} (2\pi)^{-\frac{k}{2}} \mid \sigma \mid^{-\frac{1}{2}} e^{-\frac{1}{2}(z_j - \mu_j)^T \Sigma^{-1} (z_j - \mu_j)} \\
&\times \left[2^{hk/2} \pi^{\binom{k}{2}/2} \mid g^{-1} \mid^{-h/2} \prod_{k=1}^{K} \Gamma((h + 1 - j)/2) \right]^{-1} \mid \sigma \mid^{-(h+k+1)/2} e^{-tr(g^{-1}\sigma^{-1})/2} \\
&\propto \mid \sigma \mid^{-\frac{J}{2}} e^{-\frac{tr(g_\theta \sigma^{-1})}{2}} \times \mid \sigma \mid^{-(h+k+1)/2} e^{-tr(g^{-1}\sigma^{-1})/2} \\
&\propto iWishart([g + g_\theta]^{-1}, h + J) \\
g_\theta &= \sum_{j=1}^{J} (z_j - \mu_j)^T (z_j - \mu_j)
\end{aligned} \tag{8}$$

Conjugate Gaussian priors are given to the latent peptide feature means, with each latent peptide feature sharing the same hyperparameters, and an inverse-Wishart prior to the universal component covariance. As in [?] the prior for a single assignment indicator given the rest is:

$$p(c_{d,i} = j \mid c_{-(d,i)}, \alpha) = \frac{n_{-(d,i),j} + \alpha/J}{N - 1 + \alpha} \tag{9}$$

Where $-(d,i)$ indicates all indices from all LC-MS runs except d, i, and $n_{-(d,i),j}$ is the number of measurements besides measurement i, in sample d assigned to latent peptide feature j. To prevent multiple features from the same run being assigned to the same latent peptide feature, we impose a restriction on measurement

assigment to latent peptide features such that only one measurement per LC-MS run may be assigned to a given latent peptide feature, making the conditional prior for a single assignment indicator given the rest:

$$p(c_{d,i} = j \mid c_{-(d,i)}, \alpha) = \frac{n_{-(d,i),j} + \alpha/J}{N - 1 + \alpha} \times I(\nexists\, c_{d,-i} = c_{d,i}) \tag{10}$$

where $c_{d,-i}$ indicates all indices except i from LC-MS run d. If we consider the case where the number of latent peptide features is unknown, we take the limit as J approaches ∞. Taking the limit for the conditional prior distribution on the latent peptide feature assignment indicators yields:

$$p(c_{d,i} = j \mid c_{-(d,i)}, \alpha) = \frac{n_{-(d,i),j}}{N - 1 + \alpha} \times I(\nexists\, c_{d,-i} = c_{d,i}) \tag{11}$$

$$p(c_{d,i} \neq c_{-(d,i)} \mid c_{-(d,i)}, \alpha) = \frac{\alpha}{N - 1 + \alpha} \tag{12}$$

Where 11 represents the prior probability of introducing a new latent peptide feature. Combining these priors with the likelihood conditioned on the assignment indicators, we obtain the following conditional posteriors on peptide feature assignment:

$$\begin{aligned} P(c_{d,i} = j \mid -) \propto \frac{n_{-(d,i),j}}{N - 1 + \alpha} &\times \mid (\beta_d^T \sigma \beta_d + \Sigma) \mid^{-\frac{1}{2}} \\ &\times e^{-\frac{1}{2}(x_{d,i} - \eta_d - \mu_{c_{d,i}}\beta_d)^T (\beta_d^T \sigma \beta_d + \Sigma)^{-1} (x_{d,i} - \eta_d - \mu_{c_{d,i}}\beta_d)} \\ &\times I(\nexists\, c_{d,-i} = c_{d,i}) \end{aligned} \tag{13}$$

$$\begin{aligned} P(c_{d,i} \neq c_{-(d,i)} \mid -) \propto \frac{\alpha}{N - 1 + \alpha} &\times \mid (\sigma + \beta_d^{-1} \Sigma (\beta_d^{-1})^T) \mid^{-\frac{1}{2}} \mid r \mid^{-\frac{1}{2}} \\ &\times \mid (r^{-1} + (\sigma + \beta_d^{-1} \Sigma (\beta_d^{-1})^T)^{-1}) \mid^{-\frac{1}{2}} \\ &\times e^{-\frac{1}{2}\left((x_{d,i} - \eta_d)\beta_d^{-1}\right)^T \left(\sigma + \beta_d^{-1} \Sigma (\beta_d^{-1})^T\right)^{-1} (x_{d,i} - \eta_d)\beta_d^{-1} + \lambda^T r^{-1} \lambda - B^T A^{-1} B} \\ A &= (r^{-1} + (\sigma + \beta_d^{-1} \Sigma (\beta_d^{-1})^T)^{-1}) \\ B &= (r^{-1}\lambda + (\sigma + \beta_d^{-1} \Sigma (\beta_d^{-1})^T)^{-1} [x_{d,i} - \eta_d] \beta_d^{-1}) \end{aligned} \tag{14}$$

The computation of 14 is possible because we assume all latent peptide features share the same covariance. We now describe the extension of the model to incorporate product ion spectra. To incorporate product ion data, we select up to the 50 most intense product ions for each peptide feature measurement, $x_{d,i}$. We then generate a K-dimensional product ion intensity profile for each $x_{d,i}$. Each position, y_{d,i_k}, in the product ion intensity profile, $y_{d,i}$, is computed as:

$$y_{d,i_k} = \frac{\sum_p \Omega_p \times I(M_p \leq B_k)}{\sum_p \Omega_p} \tag{15}$$

where $k = 1 \ldots K$, $p = 1 \ldots 50$, Ω is a 50-dimensional vector of intensities, M is a 50-dimensional vector of product ion mass-to-charge ratios, and B is a K-dimensional vector of product ion profile mass-to-charge ratio bin upper limits. All values in $y_{d,i}$ sum to one. The mass-to-charge ratio ranges, or bins, are determined at the initialization of the alignment, such that bin boundaries fall on mass-to-charge ratio "deserts". See section 2.1.2.3 and Figure **??** for a detailed description of bin boundary determination. To assess the similarity of a measured product ion profile and a latent product ion profile, w_j for latent peptide feature z_j, we introduce a similarity score, ψ , which is computed as the sum of squared differences of the two product ion intensity profiles, and is assumed to have an exponential distribution to encourage distances close to zero.

$$\begin{aligned} \psi_{d,i} &= (y_{di} - w_{c_{d,i}})^T (y_{d,i} - w_{c_{d,i}}) \\ \psi_{di,j} &\sim Exponential(\gamma) \end{aligned} \tag{16}$$

We assign a conjugate gamma prior to the rate parameter. The hyperparameters for profile scores are set to one.

$$\gamma \sim Gamma(a_0, b_0) \tag{17}$$

The likelihood for the product ion component of the alignment model is expressed as:

$$P(Y \mid -) = \prod_{d=1}^{D} \prod_{i=1}^{N_d} Exponential((y_{di} - w_{c_{d,i}})^T (y_{d,i} - w_{c_{d,i}}) \mid \gamma) \tag{18}$$

At each iteration of the MCMC, the product ion profile, w_j, of an existing latent peptide is updated empirically. The latent product ion profile is set to the average of the measured product profiles assigned to that latent peptide feature. The latent product ion profile, w_0, of a new latent peptide (one that currently does not exist) is a blank profile - a uniform vector of size K with each element having value 1/K. We update the rate parameter of the distribution on the similarity score as follows:

$$\begin{aligned} P(\gamma \mid \psi, a_0, b_0) &\propto P(\psi \mid \gamma) \times p(\gamma \mid a_0, b_0) \\ &\propto \prod_{d=1}^{D} \prod_{i=1}^{N_d} Exponential(\psi_{d,i} \mid \gamma) \times Gamma(\gamma \mid a_0, b_0) \\ &\propto \prod_{d=1}^{D} \prod_{i=1}^{N_d} \gamma e^{-\gamma \psi_{d,i}} \times \frac{1}{b_0^{a_0}} \frac{1}{\Gamma(a_0)} \gamma^{a_0 - 1} e^{-\frac{\gamma}{b_0}} \\ &\propto Gamma\left(a_0 + N, b_0 + \sum_{d=1}^{D} \sum_{i=1}^{N_d} \psi_{d,i}\right) \end{aligned} \tag{19}$$

Combining the product ion model with the peptide-level model, we have the following conditional posterior:

$$
\begin{aligned}
P(c_{d,i} = j \mid -) \propto \frac{n_{-(d,i),j}}{N-1+\alpha} & \times \mid (\beta_d^T \sigma \beta_d + \Sigma) \mid^{-\frac{1}{2}} \\
& \times e^{-\frac{1}{2}(x_{d,i} - \eta_d - \mu_{c_{d,i}} \beta_d)^T (\beta_d^T \sigma \beta_d + \Sigma)^{-1} (x_{d,i} - \eta_d - \mu_{c_{d,i}} \beta_d)} \\
& \times I(\nexists\, c_{d,-i} = c_{d,i}) \\
& \times \gamma e^{-\gamma (y_{di} - w_{c_{d,i}})^T (y_{d,i} - w_{c_{d,i}})}
\end{aligned}
\tag{20}
$$

$$
\begin{aligned}
P(c_{d,i} \neq c_{-(d,i)} \mid -) \propto \frac{\alpha}{N-1+\alpha} & \times \mid (\sigma + \beta_d^{-1} \Sigma (\beta_d^{-1})^T) \mid^{-\frac{1}{2}} \mid r \mid^{-\frac{1}{2}} \\
& \times \mid (r^{-1} + (\sigma + \beta_d^{-1} \Sigma (\beta_d^{-1})^T)^{-1}) \mid^{-\frac{1}{2}} \\
& \times e^{-\frac{1}{2}\left((x_{d,i} - \eta_d)\beta_d^{-1}\right)^T \left(\sigma + \beta_d^{-1} \Sigma (\beta_d^{-1})^T\right)^{-1} (x_{d,i} - \eta_d)\beta_d^{-1} + \lambda^T r^{-1} \lambda - B^T A^{-1} B} \\
& \times \gamma e^{-\gamma (y_{di} - w_0)^T (y_{d,i} - w_0)} \\
& A = (r^{-1} + (\sigma + \beta_d^{-1} \Sigma (\beta_d^{-1})^T)^{-1}) \\
& B = (r^{-1}\lambda + (\sigma + \beta_d^{-1} \Sigma (\beta_d^{-1})^T)^{-1} [x_{d,i} - \eta_d] \beta_d^{-1})
\end{aligned}
\tag{21}
$$

3 Exploration of Other HE Models

We explored additional values of K, as well as implementations of different product ion models. To assess the utility and computational complexity of various product ion models, we utilized shared identifications having peptide score 5 or greater, among the first two replicates of the *E. coli* Lysate data. We constructed product ion profiles of various sizes, K = {50, 100, 250, 500}, and assessed correct matches, nearby incorrect matches, random incorrect matches, a blank profile, and an empirical profile using eight different scoring schemes. Correct matches are shared identifications, nearby incorrect matches are the 3 closest peptides in 3-dimentionsional space (m/z, retention time, drift time) excluding the correct match, random incorrect matches are a random peptide excluding the correct match and nearby incorrect matches, a blank profile is a uniform vector of size K with each element having value 1/K, and an empirical profile is the mean of all measured product ion profiles. The eight metrics assessed were dot product, 1-norm, 2-norm, Pearson correlation, Spearman correlation, Kendall correlation, K-dimensional multivariate normal PDF, and the sum of squared differences. For each metric, we examined box plots of the scores from different match types, and measured the CPU time it took to compute the scores for all match types. In order to the alignment model to perform as desired – that is to make correct matches, and avoid incorrect matches – the appropriate metric should favor correct matches above incorrect matches and new latent peptides (blank and empirical profiles), but favor new latent peptides above incorrect matches. Figure 1 shows boxplots from three of the

metrics for two different profile sizes, illustrating different scenarios.

We see that in all three of the presented metrics, correct matches are favored above incorrect matches (larger values in the dot product and multivariate normal PDF, closest to zero in the sum of squared differences). However, in the dot product and multivariate normal box plots, we see that the incorrect matches appear to be more favorable than the addition of a new latent peptide. This would be detrimental to our alignment results by encouraging mismatches. The correlation coefficients and norms gave similar results. The sum of squared differences metric gives us a desirable result, where correct matches and the addition of new latent peptides are more favorable than incorrect matches. With regard to product ion profile size, we saw only minor differences in performance – with regards to match scores and compute times (Figure 2). We decided to use K = 250 for our analyses to minimize the potential for overlapping product ions within a single peptides' product ion profile, without a drastic increase in computational overhead.

4 Supplemental Results

4.1 Supplemental Results for E. coli Lysate Alignment

The recall rates and number of mismatches for the *E. coli* alignment have been presented considering identifications having peptide score 5 or greater. To assess the significance of the differences in these measures, we performed a series of two-sample t-tests assuming equal variance for the recall rates and incorrect matches to assess differences between alignments. The resulting p-values across the range of match probability stringencies are shown in Supplemental Tables 1 and 2.

Figures 3, 4, 5, and 6 show the recall rates and mismatch counts when considering identifications having peptide score 6 or greater, and 7 or greater, respectively. The results considering identifications of higher stringencies are consistent with those considering identifications having peptide score 5 or greater.

4.2 Supplemental Results for Decoy Experiment

We presented the results of an alignment of two technical replicates of human plasma samples with an *E. coli* lysate decoy. Figure 7 shows the results of the inverse decoy analysis (aligning technical replicates of *E. coli* lysate with a Human plasma decoy).

4.3 Supplemental Results for Identification Carryover

By aligning datasets from different human tissues, we were able to infer identifications for several of the top peptides exhibiting differential expression for a phenotype of interest – treatment response for the hepatitis-C

data. The lists of proteins inferred by this analysis are shown in Supplemental Table 3. We searched for functional enrichment of these proteins using GATHER and DAVID. The GATHER Gene Ontology results for this protein set is shown in Supplemental Table 4. The DAVID Biological Process Gene Ontology results are shown in Supplemental Table 5. In addition, the GATHER chromosome location enrichment results for the Hepatitis-C inferred protein set are shown in Supplemental Table 6.

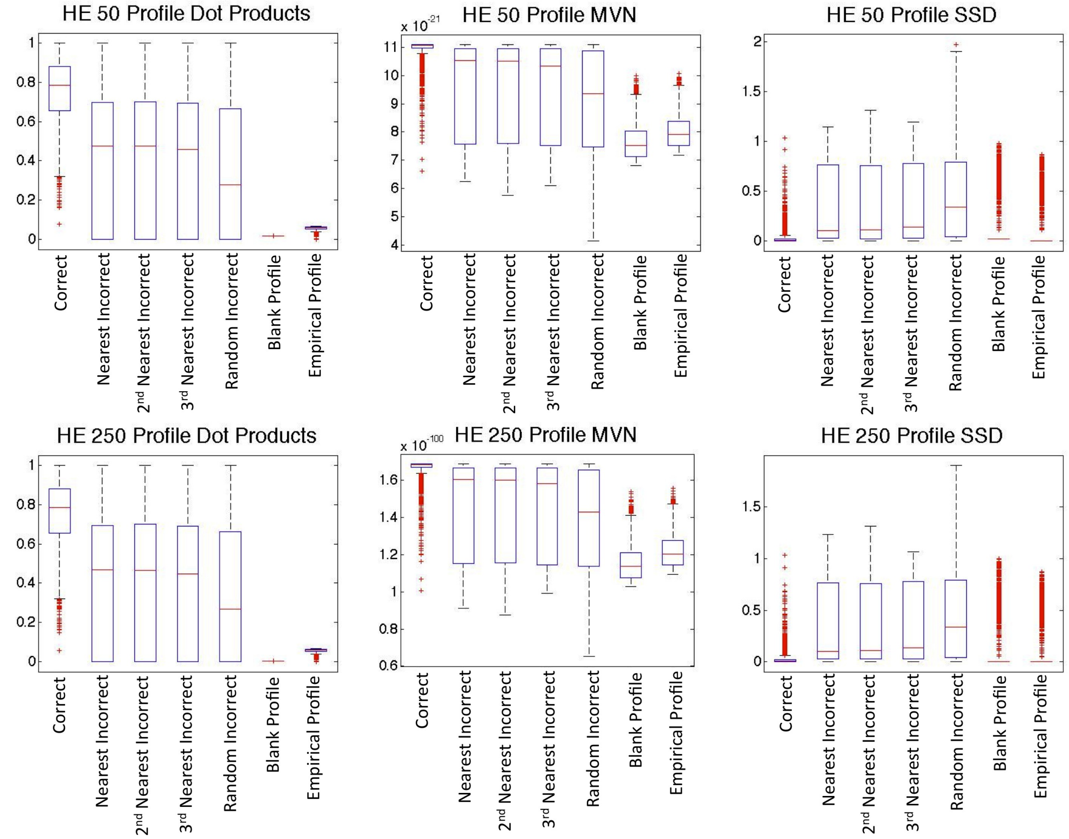

Figure 1: Results of Additional Product Ion Model Assessment. This figure shows boxplots of match scores from the Dot Product, Multivariate Normal PDF (MVN) and the sum of squared differences (SSD) metrics for two different profile sizes.

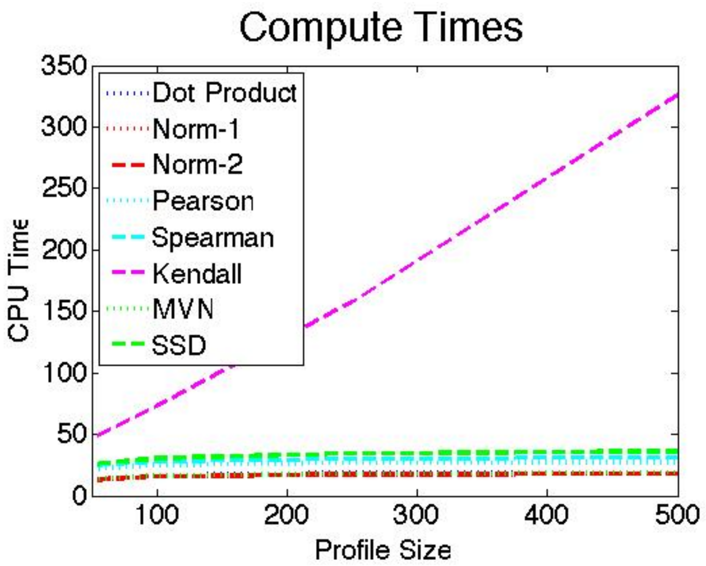

Figure 2: Compute Times from Product Ion Model Assessment. This figure shows the CPU time it took to compute the scores for the various metrics: Dot Product, 1-Norm, 2-Norm, Pearson Correlation, Spearman Correlation, Kendall Correlation, Multivariate Normal PDF (MVN) and the sum of squared differences (SSD) metrics for different profile sizes.

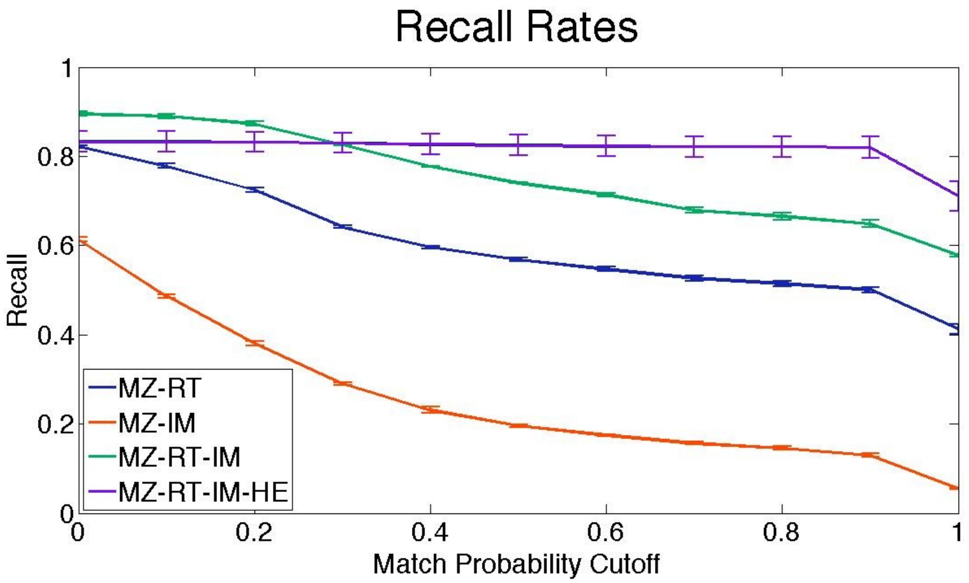

Figure 3: Recall Rates for *E. coli* Lysate Data. This figure shows the recall rate considering identifications having peptide score 6 or greater, for each of the four alignments across a range of match probability cutoffs.

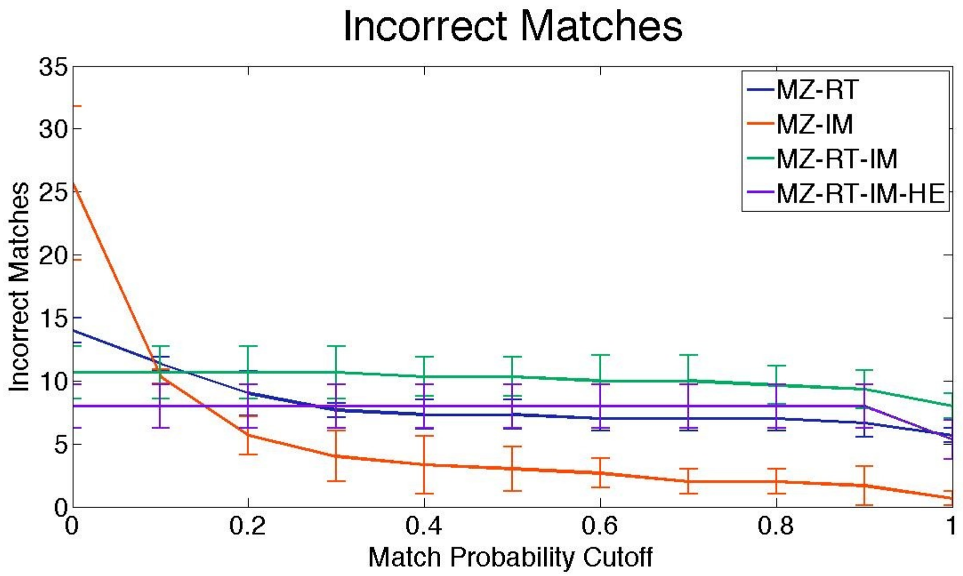

Figure 4: Incorrect Matches for *E. coli* Lysate Data. This figure shows the number of incorrect matches considering identifications having peptide score 6 or greater, for each of the four alignments across a range of match probability cutoffs.

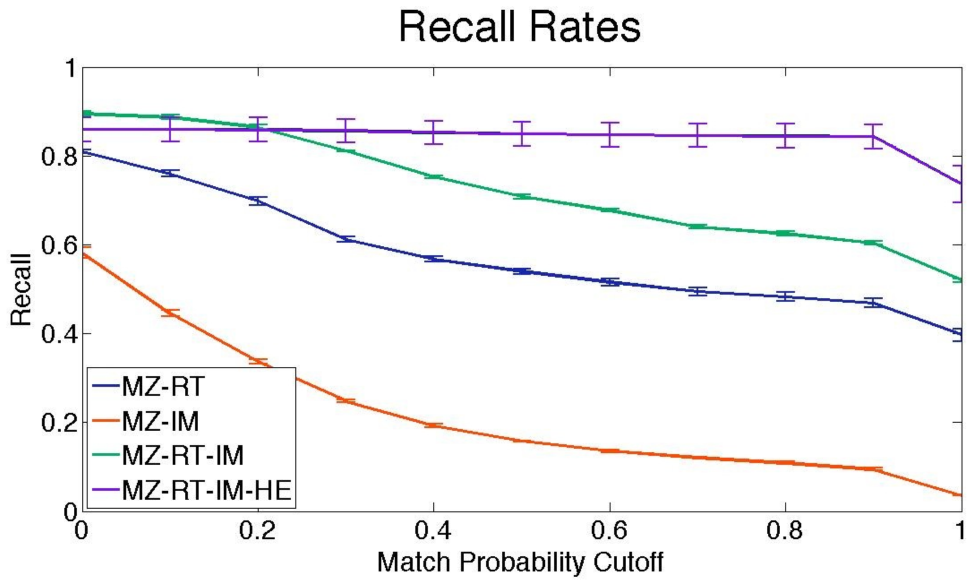

Figure 5: Recall Rates for *E. coli* Lysate Data. This figure shows the recall rate considering identifications having peptide score 7 or greater, for each of the four alignments across a range of match probability cutoffs.

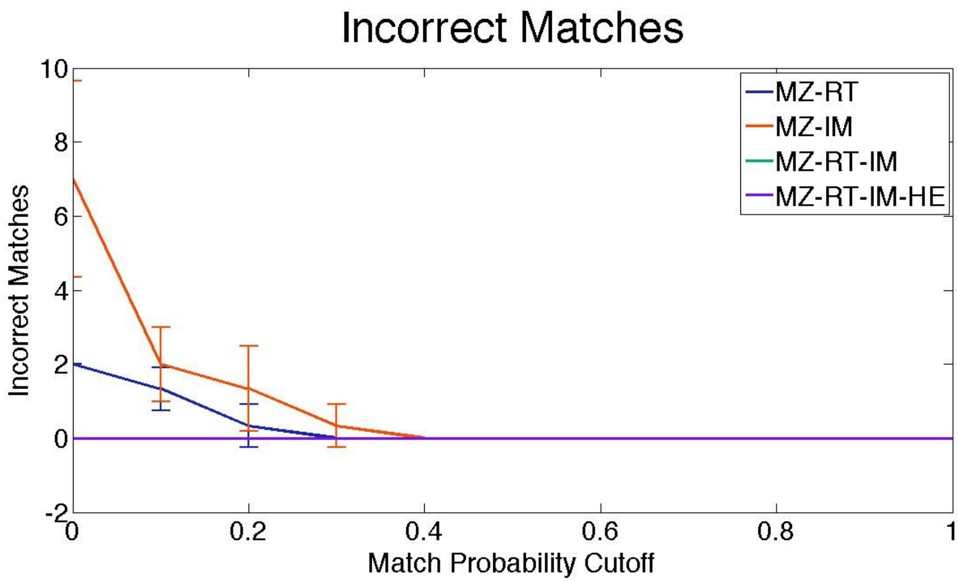

Figure 6: Incorrect Matches for *E. coli* Lysate Data. This figure shows the number of incorrect matches considering identifications having peptide score 7 or greater, for each of the four alignments across a range of match probability cutoffs.

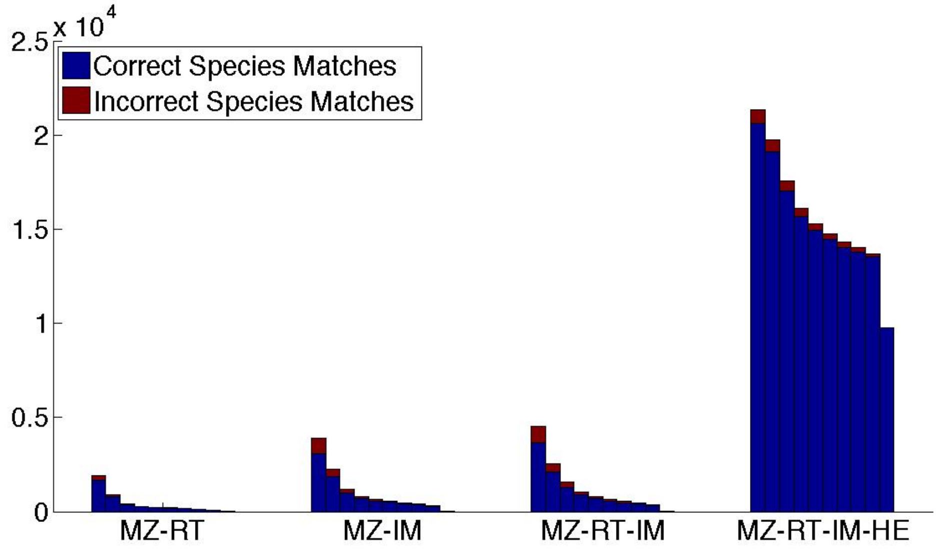

Figure 7: Correct and Incorrect Matches for Decoy Analysis. This figure shows the number of matches made to the correct species (*E. coli*), and the number of matches made to the incorrect species (Human) for each of the four alignments, across increasing match confidence thresholds from 0.1 to 1 in 0.1 intervals.
